# Temporal and spatial expression of polygalacturonase gene family members reveals divergent regulation during fleshy fruit ripening and abscission in the monocot species oil palm

**DOI:** 10.1186/1471-2229-12-150

**Published:** 2012-08-25

**Authors:** Peerapat Roongsattham, Fabienne Morcillo, Chatchawan Jantasuriyarat, Maxime Pizot, Steven Moussu, Dasuni Jayaweera, Myriam Collin, Zinnia H Gonzalez-Carranza, Philippe Amblard, James W Tregear, Somvong Tragoonrung, Jean-Luc Verdeil, Timothy J Tranbarger

**Affiliations:** 1 Institut de Recherche pour le Développement, IRD Centre de Montpellier, IRD/CIRAD Palm Development Group, DIADE 911 avenue agropolis BP 64501, 34394, Montpellier cedex 5, France; 2 Centre de Coopération Internationale en Recherche Agronomique pour le Développement, CIRAD, UMR DIADE, Montpellier, F-34398, France; 3 Department of Genetics, Faculty of Science, Kasetsart University, Bangkhen Campus, 50 Phahonyothin Road, Jatujak, Thailand; 4 Division, Loughborough, The University of Nottingham, Sutton Bonington Campus, School of Biosciences, Plant Science, Leicestershire, LE12 5RD, United Kingdom; 5 PalmElit SAS, Montferrier-sur-Lez, F–34980, France; 6 Genome Institute, National Center for Genetic Engineering and Biotechnology, BIOTEC, 113 Thailand Science Park, Phahonyothin Road, Klong 1, Klong Luang, Pathumthani, 12120, Thailand; 7 Centre de Coopération Internationale en Recherche Agronomique pour le Développement CIRAD, UMR AGAP, MRI-PHIV, Montpellier, F-34398, France

**Keywords:** Abscission, Fruit development, *Elaeis guineensis*, Polygalacturonase, Ethylene, Cell separation

## Abstract

**Background:**

Cell separation that occurs during fleshy fruit abscission and dry fruit dehiscence facilitates seed dispersal, the final stage of plant reproductive development. While our understanding of the evolutionary context of cell separation is limited mainly to the eudicot model systems tomato and *Arabidopsis*, less is known about the mechanisms underlying fruit abscission in crop species, monocots in particular. The polygalacturonase (PG) multigene family encodes enzymes involved in the depolymerisation of pectin homogalacturonan within the primary cell wall and middle lamella. PG activity is commonly found in the separation layers during organ abscission and dehiscence, however, little is known about how this gene family has diverged since the separation of monocot and eudicots and the consequence of this divergence on the abscission process.

**Results:**

The objective of the current study was to identify PGs responsible for the high activity previously observed in the abscission zone (AZ) during fruit shedding of the tropical monocot oil palm, and to analyze PG gene expression during oil palm fruit ripening and abscission. We identified 14 transcripts that encode PGs, all of which are expressed in the base of the oil palm fruit. The accumulation of five PG transcripts increase, four decrease and five do not change during ethylene treatments that induce cell separation. One PG transcript (*EgPG4*) is the most highly induced in the fruit base, with a 700–5000 fold increase during the ethylene treatment. *In situ* hybridization experiments indicate that the *EgPG4* transcript increases preferentially in the AZ cell layers in the base of the fruit in response to ethylene prior to cell separation.

**Conclusions:**

The expression pattern of *EgPG4* is consistent with the temporal and spatial requirements for cell separation to occur during oil palm fruit shedding. The sequence diversity of PGs and the complexity of their expression in the oil palm fruit tissues contrast with data from tomato, suggesting functional divergence underlying the ripening and abscission processes has occurred between these two fruit species. Furthermore, phylogenetic analysis of EgPG4 with PGs from other species suggests some conservation, but also diversification has occurred between monocots and eudicots, in particular between dry and fleshy fruit species.

## Background

The shedding of plant organs is a highly coordinated developmentally regulated event that can occur in different contexts throughout the plant life cycle [1-4]. Organ shedding is important for both plant vegetative and reproductive development, including abscission of leaves, branches, whole flowers, floral parts, seeds and immaturely aborted or ripe fruit. In particular, cell separation that occurs during fleshy fruit abscission and dry fruit dehiscence facilitates seed dispersal, the final stage of reproductive development, and therefore governs important characters in many crop species. For fruit to be shed, cell separation must occur in a precise location timed to optimize dispersal under the most favourable conditions. For crop species, if fruit are shed too early or late, economic consequences can be significant. Whereas our understanding of the evolutionary context for this phenomenon is mainly limited to model systems such as tomato and *Arabidopsis*, less is known about the mechanisms underlying fruit abscission in non-model crop species in general and, monocot species in particular.

Oil palm is a tropical perennial monocotyledonous species in the family Arecaceae with an extraordinarily oil rich fleshy mesocarp, which is the number one source of edible vegetable oil worldwide. In addition, potential use of palm oil as a biofuel is predicted to cause constraints on the worldwide supply of edible palm oil and increase the pressure for higher yields and an expansion of cultivatable areas. While conventional breeding schemes have allowed increases in yield of palm oil up to 1% per year, non-synchronized ripening and subsequent shedding of the ripest fruit before harvest limit yield gains [5,6]. In addition, the difficulty to schedule regular harvests due to non-synchronized fruit shedding results in a labour intensive logistics that increases overall production costs. Furthermore, several original characters of oil palm fruit shedding warrant further detailed investigations. In particular, the two-stage process involving primary and adjacent abscission zones (AZs), plus the extraordinary low amount of methylated pectin and high levels of polygalacturonase (PG) activity, collectively suggest that divergent mechanisms may underlie the cell separation process that leads to fruit shedding in this monocotyledonous species [7-9]. Finally, the only organ observed to shed in this palm species is the ripe fruit. Flowers and immature fruitlets from many species are naturally thinned by organ abscission in response to nutritional status to optimized reproductive success, whereas this phenomenon is not observed to any extent in oil palm. Indeed, the oil palm maintains all fruit on a bunch until ripening related signalling takes place to induce ripe fruit abscission.

While examples of organ shedding in plants are diverse, the common model proposed is mainly based on studies with eudicotyledons [2,3]. Firstly, the development of the abscission zone (AZ) takes place at the base of subtending organ to be shed. Secondly, as the AZ develops, it must become competent for cell separation events required for organ abscission. Indeed, once the AZ develops, it responds differently from adjacent tissues to the signals that induce cell separation [10]. After the AZ becomes competent for separation to be induced, cellular activity, in particular the expansion of the golgi vesicles and activation of the endomembrane system with the release of hydrolytic enzymes to the apoplast leads to the degradation of the middle lamella and cell separation [11,12]. An important feature of the model is the induction of the genes encoding cell wall hydrolytic enzymes targeted to modify and degrade cell wall components for separation to occur. The expression of these genes is often induced by ethylene and inhibited by auxin, characteristics that correlate with the positive and negative effects of these hormones on the abscission process respectively [1-3]. Despite the central importance of the mechanisms that allow changes in adhesion of adjacent cells to take place with such temporal and spatial precision, our understanding of these events even in model organisms is limited.

PG gene expression and activity are common features of organ abscission, observed in bean, tomato, peach and *Sambucus nigra*[13-16]. PG activity depolymerises the homogalacturonan backbone of pectin and while PG transcripts and activity increase in various species during the abscission process, they can also be induced by ethylene or inhibited by auxin [14,15,17-22]. In tomato, there is a single PG transcript (*pTOM6,* also known as *TFPG*) expressed during fruit ripening, while up to four other PGs (*TAPG1*, *TAPG2*, *TAPG4* and *TAPG5*) are expressed in the flower/fruit pedicel AZ associated with abscission [20,21,23-27]. Interestingly, the down-regulation or knockout of *TFPG* results in a decrease in pectin depolymerisation, but surprisingly no change in fruit softening which suggests other components are involved [25,27-29]. Furthermore, down-regulation of fruit *TFPG* has no effect on the timing or rate of leaf abscission, indicating a specific function of this enzyme during fruit ripening but not organ abscission [22]. In contrast, silencing of the abscission *TAPG1* expression delays abscission and increases break strength of the AZ [30]. Overall, these experiments suggest that while PGs are important for processes during both ripening and abscission, the same genes may not be responsible and there are other factors involved in abscission. Indeed, there are up to 69 and 59 PG genes in *Arabidopsis* and rice respectively, many with overlapping expression domains [31,32]. At least four of the *Arabidopsis* genes have expression profiles correlated to cell wall loosening and cell wall dissolution events during floral organ abscission [32]. Furthermore, *ADPG1*, *ADPG2* and *QRT2* have been shown to have overlapping functions during different cell separation processes. *ADPG1* and *ADPG2* are essential for silique dehiscence, while *ADPG2* and *QRT2* contribute to floral organ abscission, and all three genes contribute to anther dehiscence, suggesting precise combinations of PG activities may be necessary during the cell separation events underlying these different processes [33].

A previous study revealed a large increase in PG activity in the oil palm AZ in the base of the fruit during cell separation events that lead to fruit abscission [7]. Our main objective in the present study was to identify PG genes that could be responsible for this activity observed during fruit shedding. We have performed a detailed expression analysis of 14 genes that encode PGs in the base of the oil palm fruit. PG sequence diversity in the fruit tissues and their profiles of expression during fruit ripening and during ethylene induced abscission contrasts with that observed in tomato, suggesting some functional divergence underlying these processes in this monocotyledonous fruit species. The results of a phylogenetic analysis of EgPG4 with PGs with known functions and/or expression profiles from various species will also be discussed in relation to divergence that may have occurred between eudicots and monocots, in particular between fleshy and dry fruit species.

## Results

### Ethylene induced oil palm fruit shedding experimental system

**Figure 1:**
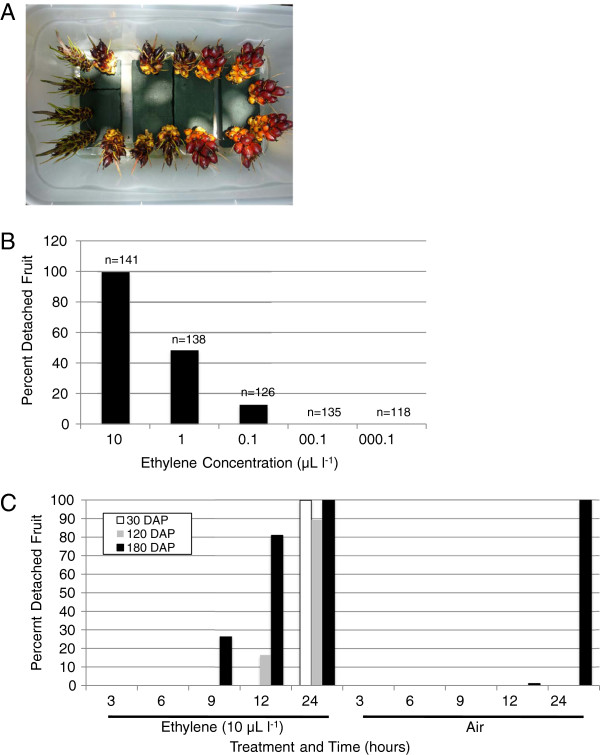
**(A) Experimental system used for ethylene-induced fruit shedding experiments.** (**B**) Dose response of ripening fruit (150 days after pollination, DAP) treated with a selected concentration range of ethylene for 12 h. (**C**) Ethylene time course treatment of oil palm fruit spikelets at contrasting stages of development (DAP 30, 120, and 180). To test for separation, fruits were subjected to light pressure and separation in the primary zone was recorded. For 30 DAP samples, n = > 200, for 120 DAP samples n = > 90 and for 180 DAP samples n = > 80 per time point/treatment respectively. Experiments were performed twice during 2010 and once during 2011 for three biological repetitions with comparable results. The data from one representative experiment are shown.

Previous studies published on oil palm fruit shedding were done with material transported by airfreight from plantations in Malaysia to a laboratory in the United Kingdom where the experiments were performed [7-9]. In order to determine precisely the timing of events that occur during abscission, our first objective was to set up an experimental system that could be used in a local field setting to eliminate problems that could arise due to the time and conditions required for storage and long distance shipment of the fruit. Based on the results of earlier studies with oil palm, ethylene was implicated as the main signal that induces cell separation in the primary AZ of the oil palm fruit [9]. Therefore, to synchronize fruit shedding, we treated spikelet explants with ethylene in airtight boxes (see Material and Methods for details; Figure 1A). The first experiment examined the ethylene dose effect on the induction of cell separation in the primary AZ of ripe fruit (150 days after pollination, DAP) treated for 12 h (Figure 1B). An increase in the number of fruit shed (13%) was observed in spikelets treated with 0.1 μl l^-1^ ethylene, while at 10 μl l^-1^, 100% of the fruit underwent cell separation in the primary AZ. This experiment confirmed the use of 10 μl l^-1^ as an effective concentration for our studies as used previously [9]. In addition, the experiment also confirmed the two-stage separation process (data not shown) during which separation first occurs within the predetermined primary AZ, followed later by separation events in adjacent AZs [8,9]. The concentration of 10 μl l^-1^ was used in further experiments to compare fruit separation at different stages of development (Figure 1C). Spikelets of fruit at 30, 120 and 180 DAP were treated and shedding was quantified at time intervals up to 24 h after treatment. No fruit were observed to shed at 3 and 6 h. Fruit at 30 DAP were only observed to shed after 24 hours of treatment, while 120 DAP fruit and 180 DAP fruit began to separate after 12 h and 9 h of treatment respectively. In air controls, only the 180 DAP fruit were observed to shed at 12 h (1%) and 24 h (100%). These experiments define the time frame during which cell separation must occur for oil palm fruit shedding to take place, and suggests an importance of developmental factors that influence the response to ethylene.

### Polygalacturonase gene family expression in the oil palm fruit tissues and the identification of the EgPG4 transcript induced in the AZ prior to fruit shedding

A 35-fold increase in polygalacturonase (PG) activity was reported to occur in the AZ during fruit shedding [7]. Furthermore, PGs are implicated in cell separation underlying organ separation in many species. In this context, our next objective was to identify PG candidate genes responsible for this large PG activity observed during cell separation events in the AZ. Briefly, our approach involved searches of available databases for sequences similar to known PGs, including locally derived 454 pyrosequencing transcriptome data, followed by designing of specific primers for each sequence identified to test, along with degenerate primers, to amplify from a mixture of cDNAs derived from fruit tissues treated or not treated with ethylene, or from genomic DNA (see Materials and Methods for details). Overall, our searches resulted in the identification of 35 putative non-redundant PG sequences, 28 of which contained either a partial or complete glycoside hydrolase family 28 (GH28) PG signature domain and were retained for further studies (see Additional file 1 for nucleotide sequences). From the 28 sequences, RT-PCR analysis revealed that 14 non-redundant PG transcripts were expressed in the AZ of oil palm fruit and a detailed analysis of their expression in fruit tissues during ethylene induced abscission was performed. The 14 transcripts are *EgPG1*, *EgPG3*, *EgPG4*, *EgPG7*, *EgPG8*, *EgPG9*, *EgPG10*, *EgPG11*, *EgPG16*, *EgPG17*, *EgPG18*, *EgPG19*, *EgPG22* and *EgPG26*.

**Figure 2:**
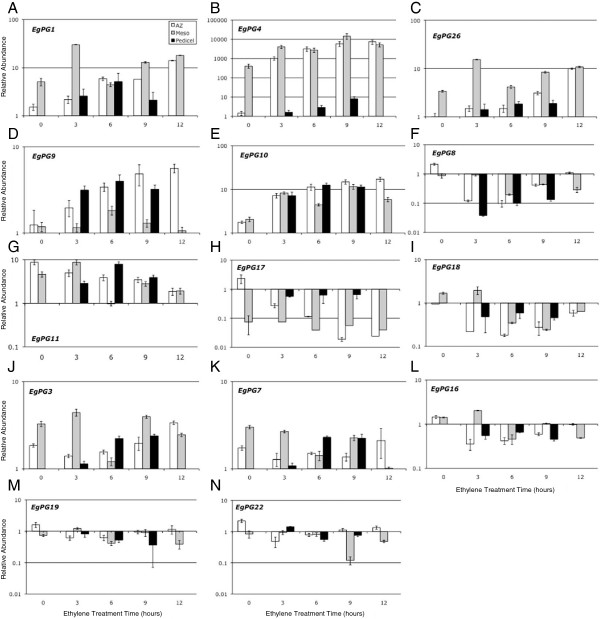
**qPCR analysis of PG transcript abundance in oil palm fruit tissues and during ethylene treatment time course.** (**A**-**E**) PG transcripts that increase during ethylene treatment in one or all tissues examined; (**F**-**I**) PG transcripts that decrease during ethylene treatment in one or all tissues examined; (**J**-**N**) PG transcripts with no significant change and/or with low abundance. Standard deviation (error bars) was calculated from three experiments. The y-axes are expressed in logarithmic scale. No data for the 12 h ethylene treated pedicel were collected.

To analyze expression, qPCR analysis was performed with tissue samples from the ethylene experiments described above (Figure 1C). The results confirmed the RT-PCR analysis in that each of the 14 primer pairs successfully amplified a PG sequence from the oil palm fruit AZ, but also from the adjacent pedicel or mesocarp tissues before and after ethylene treatment (Figure 2A-N). The profiles of transcript abundance accumulation in the AZ can be grouped into the following three main categories: I) five transcripts increase significantly (more than 2 fold; Figure 2A-E), II) four transcripts decrease significantly (more than 0.5 fold; Figure 2F-I) and, III) five transcripts have no significant change in abundance in the AZ during ethylene treatments (Figure 2J-N) respectively. By far the most abundant PG transcript detected with the most dramatic increase in abundance in the AZ is that of *EgPG4* (Figure 2B). *EgPG4* transcript increases approximately 700, 2000, 4000 and 5000 fold in the AZ after 3, 6, 9 and 12 h of ethylene treatment respectively. In contrast, *EgPG4* is also highly expressed in the mesocarp sampled from the upper portion below the apex of the untreated fruit, but only increases 10, 5, 36 and 13 fold after 3, 6, 9 and 12 h of ethylene treatment respectively (Figure 2B). Finally, *EgPG4* is faintly detectable in pedicel tissue before ethylene treatment, and increases at a lower magnitude during the ethylene treatments compared to that observed in the AZ.

**Figure 3:**
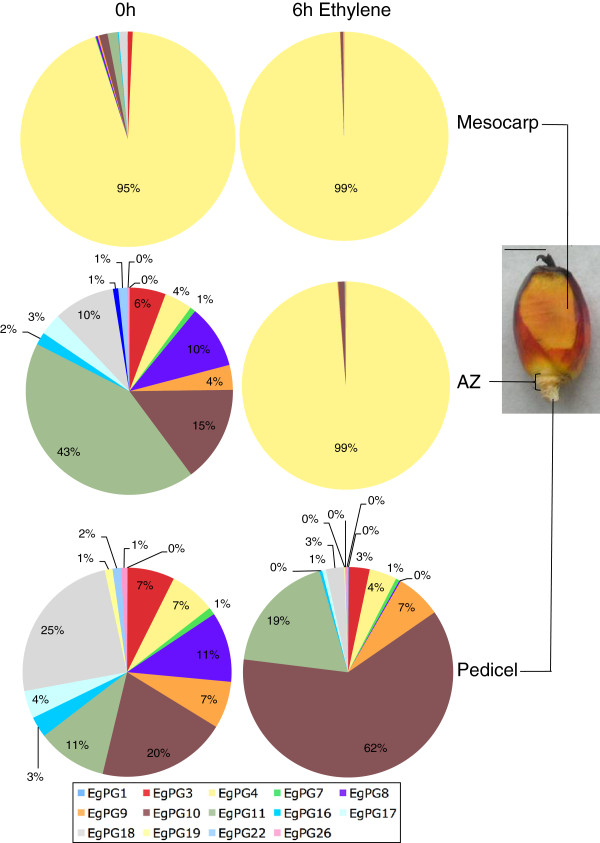
**The percentage contribution of individual PG family members to total expression in the abscission zone, mesocarp and pedicel of the oil palm fruit in untreated and 6 h ethylene treated fruit, prior to cell separation.** Data were calculated from the expression values shown in Figure 2. Standard error is in Additional file 2. Scale bar 1 cm.

An overview of PG gene expression reveals that the three adjacent fruit tissues respond differently to the ethylene treatments (Figure 3 and Figure 1 and Additional file 2). In the mesocarp below the apex of 180 DAP fruit, the *EgPG4* transcript represents 95% of the total PG transcript before ethylene treatment, then increases to 99% after 6 h of ethylene treatment (Figure 3). In contrast, in the AZ of fruit prior to ethylene treatment, *EgPG11* is the most abundant (43%) followed by *EgPG10* (15%) and *EgPG8* (10%) and *EgPG18* (10%), whereas *EgPG4*, represented only 4% of the total PG transcript detected. By contrast, *EgPG4* accounts for 99% of the PG transcript in the AZ after 6 h of ethylene treatment. In the pedicel, *EgPG10* (62%) and *EgPG11* (19%) are the most abundant PG transcripts after 6 h ethylene treatment, while the *EgPG4* transcript accounts for only 7% and 4% total transcript in untreated and ethylene treated fruit respectively. Our findings indicate that *EgPG4*, the most abundant PG transcript detected, is spatially and temporally differentially regulated in the three adjacent fruit tissues examined. Indeed, *EgPG4* accounts for the majority of the total PG transcript detected in the mesocarp, and more notably undergoes a dramatic increase in abundance preferentially in the AZ prior to the onset of separation observed after 9 h of ethylene treatment (Figure 1C).

**Figure 4:**
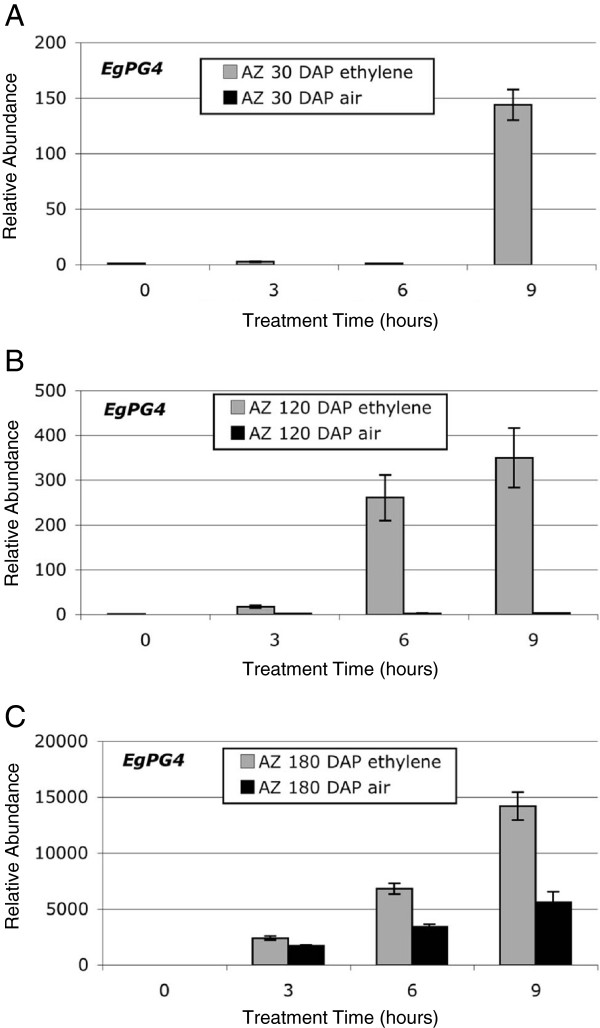
**qPCR analysis of *EgPG4 *transcript abundance in the AZ of fruit at (A) 30, (B) 120 and (C) 180 DAP treated for different time periods with ethylene (10 μl l**^**-1**^**) or air.** Standard deviation (error bars) was calculated from three experiments

During our ethylene experiments, we observed that 30 and 120 DAP fruit do not separate without treatment with ethylene (control treatments in air in the presence of ethylene absorbing material), while in the presence of ethylene they first separate after 12 h and 24 h respectively, and only after 24 h of ethylene treatment are the majority of the fruit shed (Figure 1C). By contrast, the 180 DAP fruit treated with air in the presence of ethylene absorbing material (control treatments) will begin to undergo cell separation after 12 h and will completely separate after 24 h (Figure 1C). To determine whether *EgPG4* transcript accumulation coincides with these observations, we examined the expression of *EgPG4* in 30, 120 and 180 DAP fruit in the presence or absence of ethylene (Figure 4A-C and Figure 1C). The results reveal a close correlation of the accumulation of the *EgPG4* mRNA with the timing of shedding of 30, 120 and 180 DAP fruit. Indeed, *EgPG4* has very low relative expression in untreated 30 and 120 DAP fruit compared to 180 DAP, and after 3 h of ethylene treatment, the increase is 2,400 fold in the 180 DAP fruit compared to only 2.5 fold and 17 fold in the 30 and 120 DAP fruit respectively (Figure 4A-C). After 6 h, *EgPG4* transcript increases 0.70, 260 and 6,803 fold in 30, 120 and 180 DAP fruit respectively, while after 9 h of ethylene treatment, the *EgPG4* transcript is increased 143, 350 and 14,200 fold in 30, 120 and 180 DAP fruit respectively.

### *In situ* analysis of the spatial and temporal expression of EgPG4 during ethylene induced fruit shedding

**Figure 5:**
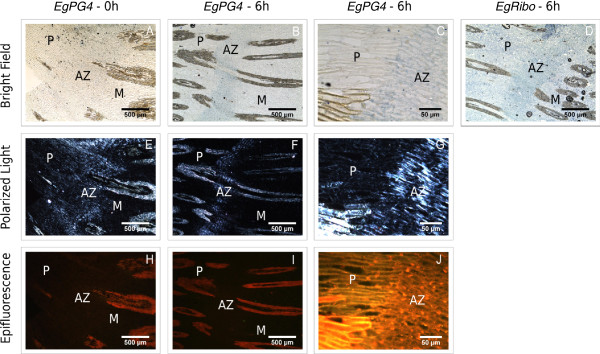
***In situ *localization of *EgPG4 *transcripts in the fruit base containing the AZ prior to cell separation.** (**A**-**C**) Longitudinal sections of the fruit base were hybridized with digoxigenin-labelled antisense RNA fragments of *EgPG4* and (**D**) the 18S ribosome and expression is observed as a blue colouring using bright field microscopy. Sections were made from fruit prior to ethylene treatment (**A**, **E**, **H**) and after 6 h of ethylene treatments (**B**-**D**, **F**, **G**, **I**, **J**). (**E**-**G**) Sections were also observed using polarized light and (**H**-**J**) epifluorescence microscopy to distinguish the AZ from the adjacent pedicel (**P**) and mesocarp (**M**) tissues.

Whereas the qPCR analysis of *EgPG4* transcript accumulation correlates well with the timing of cell separation events that occur in the base of the oil palm fruit, the AZ samples that were used for the expression analysis include a mixture of all three tissues including the AZ and the margins of the adjacent pedicel and mesocarp tissues (Figure 5). To examine whether both the temporal and spatial expression of *EgPG4* correlates with the cell separation events in the AZ that lead to fruit shedding, *in situ* hybridization analysis was performed. Firstly, we used a combination of bright field, polarized light and epifluorescence microscopy to clearly distinguish the localization of the *EgPG4* transcript within the AZ cells, compared to the adjacent mesocarp and pedicel tissues (Figure 5A-J). With polarized light, the AZ cell layers are well defined in addition to the lignified vasculature in all the tissues (Figure 5E-G). In contrast, epifluorescence microscopy mainly detected the lignified vasculature, predominantly in the pedicel and the mesocarp (Figure 5H-J). In the base of ripe fruit before ethylene treatment, the *EgPG4* transcript was neither detected in the AZ, nor in the lower margin of the mesocarp or upper margin of the pedicel tissues (Figure 5A,E,H). By 6 h after ethylene treatment, the *EgPG4* transcript increased in abundance preferentially in the AZ cell layers, including the parenchyma cells and the undifferentiated xylem cells of the vascular bundles (Figure 5B,F,I). By contrast, no *EgPG4* transcript was detected or was only present in relatively lower amounts in the adjacent pedicel and mesocarp tissues. At higher magnification of the boundary region between the pedicel and the AZ, the *EgPG4* transcript clearly accumulates in the AZ cells while it remains at very low or undetectable amounts in the adjacent pedicel cells (Figure 5C,G,J). In contrast, the control hybridizations with ribosomal RNA (rRNA) sense and antisense probes revealed a more even distribution of rRNA throughout the pedicel, AZ and mesocarp tissues when compared to *EgPG4* (Figure 5B and D; Additional file 3). Furthermore, the sense strand control with *EgPG4* also had a less intense signal than the antisense (Additional file 3). As a comparison, *in situ* hybridization experiments were also performed with *EgPG10* and *EgPG8*, the former of which is shown by qPCR analysis to increase to similar amounts in all three tissues, while the later decreases during the ethylene treatments (Figure 2E and F). For *EgPG10*, the results showed an even distribution of transcript present in the three tissues after ethylene treatment, while *EgPG8* was not detected (data not shown). Together, these results corroborate the correlation between the spatial and temporal expression profile of the *EgPG4* transcript in relation to ethylene and cell separation observed by qPCR, and provides further evidence for an important function for this transcript during fruit abscission.

### Phylogenetic analysis of EgPG4 in relation to PGs with known functions or expression profiles

To examine the relationship of EgPG4 with other plant PGs, a phylogenetic comparison of its amino acid sequence with those predicted from DNA/RNA sequences from *Arabidopsis* and rice was performed. Firstly, EgPG4 groups within the PG clade A3 formed with members from both rice and *Arabidopsis* previously defined [32] (Additional file 4). Notably, EgPG4 does not group with the PGs from *Arabidopsis* in clade A15 shown to function during floral organ abscission, silique or anther dehiscence including At2g41850 (PGAZAT/ADPG2), At3g07970 (QUARTET2), and At3g57510 (PGDZAT/ADPG1) [31-33]. However, EgPG4 is grouped in the A3 clade with two other *Arabidopsis* PGs (At2g43880 and At2g43890) that are expressed during floral organ abscission [32].

**Figure 6:**
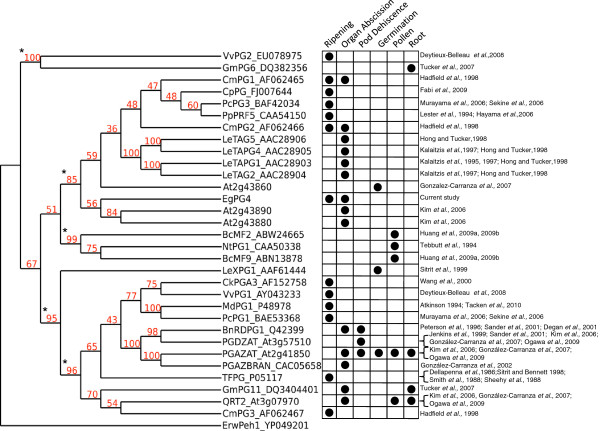
**Phylogenetic analysis of EgPG4 and selected plant PGs from clade A with known functions and/or expression profiles using the neighbor-joining method based on multiple alignment of the sequences containing the GH28 domain.** The endo-polygalacturonase ErPeh1 from *Erwinia carotovora* was used as a root. Numbers on the branches are bootstrap values for 100 replicates. The black asterisks at bootstrap values indicate branch points of the four PG subclades.

To examine possible structure-function relationships of the *EgPG4* amino acid sequence with those of known PGs from a variety of species, including those producing fleshy fruits (apple, plum, peach, tomato, kiwi, grape, papaya), and dry fruits (soybean, *B. napus*, *Arabidopsis*), a phylogenetic analysis was performed with selected plant PGs with expression associated with or shown to function during germination, root or pollen development, fruit ripening, organ abscission, and anther and pod dehiscence [19-21,23,25-27,31,33-55]. Firstly, the reconstructed tree and bootstrap values confirm earlier analyses that PGs can be separated into three major subclades, two that consist of PGs involved in fruit ripening and abscission and one with PGs involved in pollen development [18,19] (Figure 6). The presence of a fourth clade containing soybean (GmPG6_DQ382356) and grape (VvPG2_EU078975) PGs supports more recent studies that indicate this gene family consists of more than three subclades [36,56]. In addition, the bootstrap analysis confirms a close phylogenetic relationship between EgPG4 and two *Arabidopsis* PGs expressed during floral organ abscission [32]. Notably, in the same subclade there are also four abscission related tomato PGs (TAPG1, TAPG2, TAPG4 and TAPG5) [20,21,38] in addition to two PGs expressed during ripening and abscission of melon (CmPG1 and CmPG2) [19], and PGs expressed during ripening of papaya (CpPG) [37], pear (PcPG3) [53,54] and peach (PpPRF5) [42]. An additional *Arabidopsis* PG (At2g43860) that functions in cell separation between endosperm cells when the radicle emerges during germination [31] was also found within this subclade. The analysis also revealed that the *Arabidopsis* PGs involved in abscission or dehiscence including PGDZAT, PGAZAT, and QTR2, are grouped within a distinct subclade with other PGs that function during fruit ripening, floral organ abscission and pod dehiscence [31,33,35]. Notably, no PG involved in abscission of a fleshy fruit is found in this clade, only those from species with dry fruit such as *Arabidopsis*, *B. napus* and soybean.

## Discussion

### Sequence and expression analysis of EgPG4 suggests functional conservation and divergences between monocots and eudicots

PGs are thought to play a central role in the disassembly of pectin in the middle lamella or primary cell wall during cell separation and cell elongation [18]. In particular, PGs have been extensively studied during fruit ripening, organ abscission and pollen development, yet how divergence has occurred between species in order to fulfil different roles in these various tissues is still not completely understood. In particular, very little data is available from monocot species to compare the ripening and abscission processes with those of eudicots.

Our phylogenetic and bootstrap analyses confirmed several previous phylogenetic studies that revealed PGs involved in organ abscission and fruit ripening to be found mainly in two distinct subclades, with a third subclade containing only pollen related PGs [18,19,31,32]. A fourth minor subclade that contained a PG from ripening grape skin was also observed as previously [36]. While the current phylogenetic analysis was done with the complete GH28 domain, not including the prosequences characteristic of some PGs, the results support a previous conclusion that the presence or lack of prosequences are not the basis for the divergence of these sequences into distinct clades [18]. The first notable observation is that all the known tomato abscission PGs group closely within a subclade that also contains the two closely related *Arabidopsis* floral organ abscission associated PGs (At2g43890 and At2g43880), in addition to an *Arabidopsis* PG (At2g43860) implicated in radicle emergence [31,32]. The presence of At2g43860, a PG expressed during the separation of endosperm cells when the radicle emerges during germination, suggests structural relationships between PGs with functions beyond fruit ripening and organ abscission. It is interesting that EgPG4 groups closest with these *Arabidopsis* abscission PGs, which suggests functional conservation, and that these sequences are derived from a common ancestral PG that existed prior to the separation of monocots and eudicots. A second notable observation is that the tomato fruit PG (TFPG) is more closely associated with the abscission and dry fruit dehiscence PGs than with the tomato PGs involved in organ abscission. This contrasts with sequences from melon that are in the same subclade as the tomato abscission PGs, and have expression profiles associated with both fruit ripening and abscission [19]. Similarly, the *EgPG4* transcript does not only increase in the AZ in relation to abscission, but also is highly expressed in the portion of the ripening mesocarp. Together, it appears that some PGs may function both in ripening fruit tissues, in addition to during cell separation in the AZ that leads to fruit organ abscission in monocots and eudicots.

### The sequence and expression of EgPG4 suggest functional divergence between dry and fleshy fruit

Another notable observation is that PGs related to fleshy fruit abscission are not found within the clade containing the well-characterized abscission and/or dehiscent related PGs including QRT2, PGAZAT, PGDZAT, BnRDPG1 and PGAZBRAN [33,34]. By contrast, PGs involved in fleshy fruit ripening, such as the tomato (TFPG), grape (VvPG1), apple (MdPG1), kiwi (CkPGA3) and pear (PcPG1) are also found within this clade [23,25-27,36,43,44,48,50,51]. In addition, only the melon PG (CmPG3) that has an expression profile related to ripening is found in the same subclade as the dry fruit dehiscence and abscission PGs, while the other two melon PGs (CmPG1 and CmPG2) associated with organ abscission and ripening are in the same subclade as EgPG4 [19]. While there is no current data that suggests that the fleshy fruit PGs within this subclade are involved in fruit or other organ abscission, it is possible their involvement in cell separation during organ abscission has not been sufficiently investigated. Indeed, the analysis and results discussed here are based on the two best-characterized organ abscission model systems available, namely tomato and *Arabidopsis*, and it should be emphasised that many gaps exist in our current knowledge about the functional diversity of plant PGs. Nevertheless, the results suggest that dry fruit species may have PGs from at least two divergent subclades involved in cell separation for dehiscence, while fleshy fruit may have PGs specialized in ripening or abscission, or, that may function in both contexts. Overall, the results suggest that divergence may have occurred between PGs involved in dry fruit dehiscence and fleshy fruit abscission, an area that merits further investigation.

### The high expression and induction of EgPG4 by ethylene suggests functions during both fruit ripening and abscission

The most notable result of this study is the high accumulation of the *EgPG4* transcript in the base of the fruit containing the AZ prior to cell separation. Importantly, *EgPG4* transcript accumulates prior to the occurrence of cell separation, and also accumulates less and in correlation to the timing of the slower separation in fruit at earlier stages of development. However, the *EgPG4* transcript is also highly expressed in the mesocarp tissue near the apex of the fruit that suggests a role in the ripening of this tissue. Our data also indicate that the regulation of *EgPG4* is closely associated with the capacity for cells to respond to ethylene. This in turn is related to the developmental stage of ripening, and may be an important factor that controls the spatial and temporal functionality of *EgPG4* during mesocarp ripening and cell separation in the AZ. Indeed, the mesocarp produces an increasing amount of ethylene during ripening, and production progresses from the apex of the fruit to the fruit base, where it may act as the signal to initiate the separation events within the AZ [9,57]. Studies on fruit ripening and floral pedicel abscission of tomato provide examples of how individual members of this gene family may have distinct functions in adjacent tissues undergoing cell separation processes in a fleshy fruit species, and highlight the central importance of tissue specific transcriptional regulation of PGs during these developmental processes. Indeed, the tomato fruit *TFPG* is the only PG gene expressed in the ripening fruit tissues, its transcription is positively regulated by ethylene, and the encoded protein is responsible for the PG activity required for pectin depolymerisation that occurs during ripening [23-27]. Notably, the *TFPG* mRNA accounts for up to 2.3% of the total RNA in ripening tomato fruit, and down regulation of *TFPG* has no effect on the timing or rate of leaf abscission, indicating a specific function of this enzyme during fruit ripening but not organ abscission [22,58]. In contrast, in the pedicel where the AZ is located at the base of the tomato floral organs, there are at least four abscission-related PG genes (*TAPG1*, *TAPG2*, *TAPG4* and *TAPG5*) expressed, three of which are induced by ethylene and correlate well with the cell separation that occurs in the flower and leaf AZs [20,21,38]. Furthermore, silencing of the tomato abscission-related PGs using a *TAPG1* fragment, delayed abscission and increased break strength of the leaf petiole AZs in explants treated with ethylene. These studies suggest that a combination of tissue specific transcriptional regulation and/or localized cellular differences in response to ethylene are important factors that determine the spatial and temporal specificities related to their functional roles during fruit ripening and organ abscission.

Oil palm fruit shedding has some similarities but also notable differences from that seen in tomato. Firstly, the timing of separation induced by ethylene in oil palm is comparable to that in tomato. In the presence of ethylene, cell separation begins to occur by 9 h, while 80–100% of ripe fruit are shed by 12 h, whereas in tomato, flower shedding begins at 6 h and is complete by 12 h [59]. This result is striking given the surface area of the primary AZ of ripe oil palm, up to 10 mm (Figure 3) is approximately 20 times larger than the tomato pedicel AZ, up to 0.55 mm [59]. Secondly, we observe a greater diversity of *PGs* expressed in the oil palm fruit tissues than that of tomato during ripening or abscission. Notably, of the 14 transcripts expressed in the base of the fruit containing the AZ, five are regulated positively, and four others negatively in response to ethylene. In addition, five PG mRNAs displayed no significant change in abundance during the ethylene treatments. A previous study with banana fruit revealed that at least four PG genes are expressed during ripening [60]. However, none of the PGs identified in that study contained the full-length GH28 domain and thus we were not able to compare their phylogenetic relationship with the oil palm PGs and other PGs presented in Figure 6. The expression of the banana PG genes was also analyzed during finger drop, a process that also involves pectin disassembly [60,61]. The results indicated that the four banana PGs were also expressed in the finger drop zone where cell separation takes place, while *MaPG4* was the most highly expressed with a profile of accumulation correlated to the decrease in the pedicel rupture force observed. Together with the present results, the mechanisms of pectin disassembly during banana and oil palm fruit ripening may involve a larger number of PGs than with eudicot species examined thus far. The current study allows a more complete view of PG expression in relation to ethylene in a monocot fruit, given that the earlier studies with banana included fewer and shorter PG sequences [60,61]. In addition, whereas both are monocots, the banana is a parthenocarpic berry-type fruit that accumulates large amounts of starch, while the oil palm is a drupe with the high oil content, which may also dictate different ripening regulatory mechanisms between these two species. Future work will require new molecular resources for more complete comparative studies of fruit ripening and abscission in these two diverse monocots, in addition to the well-characterized eudicot tomato model.

In comparison to tomato, the diversity and complexity of PG expression in the oil palm fruit tissues is far greater than that observed in the AZs or during ripening. In the oil palm, all 14 *EgPG* transcripts are detected to some extent in the ripening mesocarp tissue, in contrast to the single *TFPG* expressed during tomato fruit ripening. Notably, none of the *EgPGs* mRNAs identified appears to be completely tissue specific, as observed with the tomato PGs involved in abscission and ripening. However, the data presented here suggest that differences in their tissue and developmental stage dependent response to ethylene may be important for spatial and temporal control. The most notable example is that of *EgPG4*, which is not only the most abundant PG transcript in the mesocarp of untreated ripe fruit, but also undergoes the most dramatic increase in abundance in the base of the fruit containing the AZ in response to ethylene. The high abundance of *EgPG4* in the mesocarp and the massive increase in response to ethylene is similar to PG expression in tomato; however, *EgPG4* is highly expressed in both the ripening mesocarp and the AZ after ethylene treatment prior to fruit shedding. Furthermore, our *in situ* hybridization experiments indicate the increase in *EgPG4* transcript abundance in the base of the fruit occurs preferentially in the AZ compared with the adjacent mesocarp and pedicel tissues. Importantly, a delayed and less significant increase in *EgPG4* transcript is also observed in the AZ of untreated fruit, as well as in 30 and 120 DAP fruit treated with ethylene, which corresponds to the delay in shedding observed at these stages of ripening.

## Conclusions

Together, these results provide evidence that *EgPG4* participates in cell wall pectin modifications during both mesocarp ripening and in the AZ cells during fruit shedding, in close relation to a developmentally regulated cell sensitivity or competence to respond to ethylene. Future work will be aimed at identifying the regulatory factors that control the ripening and abscission related expression of *EgPG4*, to provide a basis to compare these processes not only between monocots and eudicots, but in particular between fleshy and dry fruit species. Finally, the identification of genes involved in oil palm fruit shedding will also be helpful for oil palm improvement selection strategies.

## Methods

### Plant material, ethylene treatment and RNA extraction

Oil palm (*Elaeis guineensis* Jacq) fruits were harvested at Krabi Golden Tenera plantation, from a *tenera* clone (clone C) produced in Thailand. For each stage of development studied, independent bunches were collected from distinct individuals of the same genotype. Spikelets were then collected in the centre of each bunch and sets of 6 spikelets were randomly sampled from them and put in individual hermetically sealed 50 l volume boxes. Spikelets with fruits at 150 days after pollination (DAP) were treated with different concentrations of ethylene (0, 0.001, 0.01, 0.1, 1, 10 μl l^-1^). In absence of ethylene treatment, ethylene absorber (ETHYL-GONE, http://www.biosafer.com/ethyl-gone.php) was added in the box. All the boxes were kept at ambient temperature (approximately 30°C), and after 24 h of treatment the number of fruit separating from the spikelets were counted. Using the concentration of ethylene (10 μl l^-1^) that induced and synchronized the highest amount of fruit shedding, a time course analysis was then conducted that used the same process with fruit from 30, 120 and 180 DAP. Spikelets were treated with or without ethylene, and every 3 h, treated or untreated spikelets were collected and shedding was quantified for each stage of development. For each time point, the mesocarp, pedicel and the base of the fruit containing the primary and adjacent AZs were isolated and frozen immediately in liquid nitrogen. Samples from two independent experiments were collected immediately after bunches were harvested.

Total RNA from mesocarp, pedicel and the base of the fruit enriched in AZs, treated or not with ethylene was extracted as previously described [62]. Total RNA (1 μg) was used to synthesize cDNA using the first-strand cDNA synthesis kit (ImProm-II™ Reverse Transcription System, Promega).

### Identification of oil palm non-redundant PG nucleotide sequences from fruit

To identify oil palm PG cDNA sequences a number of molecular resources were used. First, the tblastn program was used to search available databases that contain *Elaeis guineensis* sequences, including NCBI (http://www.ncbi.nlm.nih.gov), local 454 pyrosequencing derived oil palm mesocarp contigs [57] and contigs derived from tissues enriched in the AZ (Jantasuriyarat *et al.*, unpublished), for sequences with high similarity to PGs from *Arabidopsis* and rice previously described [32]. Additional sequences were also kindly contributed by Dr Arondel [63]. A complementary approach utilized degenerate primers [34] to amplify cDNAs from AZ tissues treated with or without ethylene at different developmental stages and from oil palm genomic DNA. Primers from the oil palm PEST643 (accession number N° AY291341) were designed in the most conserved regions of PGs and also used to amplify PG cDNAs from fruit tissues. For sequences lacking the 3’ regions, RACE (Clontech) amplification was performed and from sequences obtained, sequence specific primer pairs were designed and used to amplify non-redundant PGs from the oil palm fruit tissues. A total of 35 putative non-redundant PG sequences were identified from these complementary approaches and were compared to confirm similarity to plant PGs, in particular the presence of a partial or complete glycoside hydrolase 28 (GH28) domain that covers approximately 75% of each PG coding sequence [35]. The accession numbers for *EgPG1* and *EgPG4* are JX233615 and JX233616 respectively, while other PG sequences are from previous datasets [57,63].

### Quantitative Real-Time RT-PCR

qPCR was conducted on a LightCycler 480 (Roche) in 96 well plates in a volume of 10 μl containing 2 μl of cDNA diluted 1/100, 1.5 μl of primer forward (2 μM), 1.5 μl of reverse primer (2 μM) and 5 μl SYBR® Green Mastermix (Roche). Additional file 5 lists the primers used. PCR was initiated by denaturation at 95°C for 10 min, followed by 45 cycles of 95°C for 15 s, 60°C for 15 s, and a final extension at 70°C for 1 min. All expression was normalized to the *EgEfα1* (accession number: AY550990) mRNA from *Elaeis guineensis*, and relative mRNA abundance was determined with the formula as described previously [64]. No change of *EgEfα1* transcript accumulation was found in the fruit tissues treated or not treated with ethylene. Control using RNA matrices were also conducted to validate the absence of DNA in each sample. Each time point was replicated three times from 2 independent biological samples, and all amplified cDNA fragments were sequenced by Beckman-Cogenics to check the specificity of the amplified products. Gene abundance is expressed as mean and standard error bars are calculated from the technical replicates of one of the biological repetitions.

### Phylogenetic analysis

Phylogenetic trees were constructed based on similarity searches performed with BLASTp programs with default parameters in protein sequence databases provided by the NCBI server (http://www.ncbi.nlm.nih.gov). Phylogenetic analyses were performed on the Phylogeny.fr platform (http://www.phylogeny.fr) [65]. Amino acid sequences from the GH28 domain were aligned with ClustalW (v2.0.3) [66]. After alignment, ambiguous regions (i.e. containing gaps and/or poorly aligned) were removed with Gblocks (v0.91b). The phylogenetic tree was constructed using the neighbour joining method implemented in Neighbor from the PHYLIP package (v3.66) [67]. Distances were calculated using ProtDist. The Jones-Taylor-Thornton substitution model was selected for the analysis [68]. The robustness of the nodes was assessed by bootstrap proportion analysis computed from 100 replicates [69]. Graphical representation and edition of the phylogenetic tree were performed with TreeDyn (v198.3) and Inkskape respectively.

### RNA *in situ* hybridization

To obtain DNA templates for the RNA probe synthesis, PCR amplifications were performed with gene-specific antisense primers tailed with a T7 RNA polymerase binding site. PCRs were performed with the EgPG4qS1–EgPG4qAS1T7 and EgPGq4S1T7–EgPG4qAS1, and the EgRiboS-EgRiboAST7 and EgRiboST7–EgRiboAS primer pairs for *EgPG4*, and *EgRibo*-specific probes, respectively (Additional file 6). The resulting DNA fragments were used directly as templates to synthesize antisense probes, with the incorporation of UTP–digoxigenin (Roche) as the label using the MAXIscript® T7 Kit (Ambion). Each amplification product was sequenced to check the specificity of the products amplified*. In situ* hybridization experiments were carried out as described previously [70] with some modifications. The fruit bases from untreated fruits and fruits treated with 10 μl l^-1^ of ethylene for 6 h were fixed overnight in the dark at 4°C in fixation buffer (4% paraformaldehyde, 0.1 M phosphate buffer pH 7). After 16h, they were washed two times in 0.1 M phosphate buffer with 2% glycine, then two times in 0.1 M phosphate buffer before dehydration through an increasing series of ethanol and butanol concentrations. After 15 days in butanol to soften the tissues, the samples were embedded in Paraplast plus (Paraplast X-Tra, Oxford Labware) and sectioned to 12 μm with a microtome. Tissue sections were deparaffinised with Safesol (LaboNord, France), rehydrated through an ethanol series of decreasing concentrations, and then pre-treated with proteinase K (100 U μl^-1^, Roche) in Tris–HCl (100 mM, pH 7.5), EDTA (50 mM) at 37°C for 35 min. Digestion was stopped by washing twice for 5 min each with TRIS–HCL (20 mM, pH 7.5, CaCl_2_ (2mM) and MgCl_2_ (50 mM), then phosphate-buffered saline (0.1 M PBS) with 0.2% glycine for 2 min, and then twice with 0.1 M PBS. After ethanol baths, hybridization was performed at 45°C overnight with 200 ng of the digoxigenin-labelled RNA probe in 100 μl of hybridization solution (50 μl formamide, 10 μl 20X SSC, 1 μl Denhardt 100X, 20 μl dextran sulphate 50%, 1 μl tRNA at 100 mg ml^-1^). After hybridization, slides were washed in 2X SSC at 25°C for 5 min, in 2X SSC at 50°C for 45 min and in 1X NTE (Tris–HCl 10 mM, NaCl 0.5 M, EDTA 1 mM, pH 7.5) at 25°C then 37°C for 5 min each. An RNase A digestion (20 μg ml^-1^) was carried out for 30 min at 37°C and stopped by washing with 1X NTE at 37°C. Final washes were conducted twice in 1X SSC for 30 min each at 55°C. Detection was performed using the Vector Blue Alkaline Phosphatase Substrate Kit III (Vector Laboratories). Control without probe was conducted to valid the absence of endogenous alkaline phosphate activity. Samples were incubated in blocking reagent [Roche; 10% (w/v) in PBS] for 1 h and afterwards for 45 min at 37°C containing antidigoxigenin alkaline phosphatase-conjugated Fab fragment antibody (Roche) diluted at 1:500 in blocking reagent. After three washes for 10 min in 0.1 M PBS, tissues were equilibrated in detection buffer (100 mM Tris–HCl pH 8.2) then several batches of 3 h at 37°C with Blue vector. Finally the detection was amplified by ethanol vapour for 20 min and samples were mounted on slides with Mowiol and observed with a bright-field microscope (Leica DM6000) using the 40X/0.75 numeric aperture. To visualize the abscission zone, tissue sections were also observed under polarized light and epifluorescence with a TXR filter. Photographs were taken with a Retiga 2000R camera (Qimaging). *In situ* hybridization and microscopy analysis were conducted at the “Plate-Forme d’Histocytologie et Imagerie Cellulaire Végétale” (PHIV platform; http://phiv.cirad.fr/).

## Abbreviations

PG: Polygalacturonase; AZ: Abscission zone; GH28: Glycoside hydrolase family 28; DAP: Days after pollination.

## Competing interests

There are no competing interests to declare.

## Authors’ contributions

TJT and FM devised and participated in all aspects of the study. TJT and ST coordinated the logistics for study. TJT, FM, PR, CJ and MP performed the ethylene experiments and collected samples for RNA isolation and *in situ* hybridization studies. PR extracted total RNA, isolated polygalacturonase cDNAs, performed cloning, designed gene specific primers and performed preliminary RT-PCR expression studies. ZHGC participated in the identification of putative polygalacturonase cDNAs. MP and FM performed the qPCR analysis. ST and PA participated in the data analysis and critically read the manuscript. SM and FM performed the phylogenetic analysis. JLV, SM, DJ and MC prepared samples for histological analysis and performed *in situ* hybridizations. TJT, JLV, FM, ZHGC, JWT and PR participated in writing the article. All authors read and approved the final submitted manuscript.

## Supplementary Material

Additional file 1 List of the 28 sequences that contain either a partial or complete GH28 PG signature domain. Click here for file

Additional file 2 **Standard errors for Figure **3**.** Percentages were calculated from gene expression data derived from qPCR analysis that included individual values (3 technical repetitions) compared to the average expression of the reference gene (*EgEF1α*, elongation factor 1 α), together with the standard deviation (SD) for the following three tissue regions of the fruit: AZ, Abscission Zone; M, Mesocarp; P, Pedicel. Click here for file

Additional file 3 **Control experiments for *in situ *hybridization studies.** Longitudinal sections of the fruit base were hybridized with digoxigenin-labelled RNA fragments of *EgPG4* antisense (A) and sense (B), and the 18S ribosome antisense (C) and sense (D) probes after 6h ethylene treatment. Click here for file

Additional file 4 **Phylogenetic analysis of EgPG4, EgPG8 and EgPG10 with sequences from *Arabidopsis *** **and rice.** Click here for file

Additional file 5 List of primers used for expression analysis of oil palm PG genes by qPCR. Click here for file

Additional file 6 **List of primers used for the synthesis of *in situ *** **hybridation probes.** Click here for file
