# Supplementary material for: Temporal and spatial expression of polygalacturonase gene family members reveals divergent regulation during fleshy fruit ripening and abscission in the monocot species oil palm
Source: BMC Plant Biol. 2012 Aug 25;12:150. doi: 10.1186/1471-2229-12-150 (PMC3546427; doi:10.1186/1471-2229-12-150)
Supplement: Additional file 1 — List of the 28 sequences that contain either a partial or complete GH28 PG signature domain. [file 1471-2229-12-150-S1.doc]

**Table S3 List of the 28 sequences contained either a partial or complete GH28 PG signature domain.**

| **Name of genes** | **sequence** |
| --- | --- |
| ***EgPG1*** | **>Contig_223666S6AS7**  AGGCATCAGCATTGGGAGCCTCGGCGTGCACAACTCGCAGGCTTGCGTTTCGAACATTAGGGTCAAGAATGCAGTGATCAGGAACTCTGACAATGGGGTCAGGATCAAGACATGGCAGGGTGGGATGGGCTCAGTAACAAGCATTAGCTTCGAGAACATCATCATGGAGAATGTTAAAAACTGCATCATTATAGACCAGTACTATTGCCTAAGCAAGCAGTGTATGAATCAAACGTCGGCGGTCTACGTAAAGGATGTCTCATACATGAACATAAAGGGGACGTATGATGTCAGGAGCCCACCGATACACTTTGCTTGCAGTGATGCCGTGCCATGCACCAACATCACCATGTCTGATGTGGAGCTGCTCCCATTTGATGGAGAGTTGGTGGATGACCCCTTTTGTTGGAATGCCTATGGGAGCATGGAGACACTAACAATTCCACCCATCTCTTGCTTGCAGGATGGGGAGCCTCAGTCCATCAAAGAGAACCTTAGATTAGGTTGCTGAACTTGTTAGTGCTTCAGGTACCGCAATTTAGAGACACAATAACACAAACAGAGCTAAAGACCCATATGGTCAAACCTTTATTTTTTCCAATATGTGGCTGTTGTGGGGATTTAGTCATGCTTGGGGGGCCCTCATGGAGCTTGTTTTCATGTATTGATCGGAACATATTATGCATGGGCTTATTTCAAAGTGTCAAAAGTTCAGGTAGTTTGAGTATATTTGTGCACCTGTGGAAGGAACAATGTTTGGCTCTGTTTAACTTCTGTAGCTGGTTCACTAGAAGAAACGGATAGTGACGAGAATTTTTCTGTAAAAAAAAAAAAAAAAAAAAAA |
| ***EgPG3*** | **>CL1Contig7023**  TTGTTACATCAGCACTGGTGGATGATCTAAATTGCAGTCAAAAGCGGACTGGGAGTGAATATGGCATTCCTTTGCTCATCCAAGCTCCAATATTAGTATCCGCCGCATCACTGGAGAAACAAAATTAGGTGCTGGCATTGCGTTTGGAAGTGAGATGTCTGGTGGTGTATCAGAAGTTTGGGCAGAGGACATCCACCTCTTTAACTCAAAGCATGGTATCAGAATAAAACATCTCCAGGGCGAGGTGGCTATATTCGAAATATCTACATCTCTGATGTGATCATGAAGAATGTGGACATAGCGATCAGGATCCAAGGTCAATATGGCGAGCATCCAGATGAAAAGTATGACCCAAATGCACTTCCAATCATAAACAGGATAACCATAAAAAATGTTGCGGGAGCAAATATCAGACTTGCAGGTCTTTTAGAGGGTATTCACGGAGATAATTTCAGCAACATATGCCTATCTAATATCATCCTTAACGTAACATCCCCTCATCCTTGGAATTGTTCATATGTTGAAGGTTATTCCAATATGGTGTCTCCAGAGTCCTGTGAGCCTCTTAAAGAAATACCTGAAGATTCTTCTATTTGTTACCCAGCTAATCATTTGAAACCACAACAATCCAGTGCAATTCGTTTGATTAGTCCCTTCATTAAGTTCATAGCAATGTAACCAAAGTCTGGCGCTGTATATAAGTTGGAACTTCTGTCAAGGGATCTTCATTTGTCCGAAATGTTTGGTTTTGTATGGTTTTTCAACAATAAAGTGTATAGAGTTTTGGGGTTTCCGTCATGTAAGTAATTGTATGGTGAACAGCTATATGAAATTGATTTTTTTTTTTTTCCTTGTAACATTTTTGGCAAGCAATTTATTTGAGATGAGGATCTTGC |
| ***EgPG4*** | **>CL1Contig5616**  GATGCATCCATCGAAGACGCTCTGCAGCATCACGTGAGCTGCTTCAGCGGCCATCGGAGCACTGGAATCGGAAGAAGAAGGCTTTTTGGCTCCCCCCGCGTACTCTGCGTTGATACCACTGCTTAAGCAGTGGTATCAACGCAGAGTACGCGGGGCATCCAAGTCCCTCCAAACCCACTCATACCTATATCTCATAATCTCCTTCCTTTAAGACCTTCTATACTTTCATAAAAAAAAGGAGTCCAATGACTCAAATCATGTGCTCCTTCCGCCTCCTCCTCCTAGCAATTCTCATGATCCATTTCTCCTCATCAGTAAAAGCATCTTACGACGTTACTTCCTTTGGTGCCAAACCCAATGGTCGGACAGACTCGACCAAATCATTCATTAGTGCATGGACTGCCGCCTGTCACTCAACAAACCCTGCAACCATCCATGTACCCTCCGGCTTCTTCCTGATAGGTCCGGTCACCTTCAATGGTCCTTGTAGGAGCAGTAGGATCACCATCCAGATCGACGGAACCCTCGTCGCACCATCGAATTATGCTAATCTTCGCAATCTTGAACAGTGGATTTTGTTCGATCATGTCGACGGCATCTCGGTGTACGGTGGGACCATCGACGGCCATGGCTCACCTCTATGGGCCTGCAAGGCTGCCGGCCGGAACTGCCCTGCCGGTGCAAGGTCGTTAGTGTTCAGGAACTCGAAGAACATCTTGATCAGTGGACTGACGTCGATCAATAGCGAGAGGTTCCACATCGTGATCGATGGCTGCCAGGTGGTGACGGTGCACGGGGTCAGGATCACGGCCCCCGGCAACAGCCCCAACACCGATGGCATCCACGTCCAGTCCTCCACCGACGTCACGATCACCGGAGCCGGCATCAAGACGGGCGACGACTGCATCTCGATCGGGCCCGGCACGACGAACCTATGGATCGAGAAGGTCACTTGTGGACCTGGCCATGGCATAAGCATTGGGAGCTTGGGGAATGAGTACGAGGAGAAGGGGGTGGAGAACGTAACGGTGAAGACGGCGGTGTTCACGGGAACGGAGAACGGGCTGAGGATAAAGACGTGGGGGAGGCCGAGCGAGGGGTTCGTGAAGGGAGTGGTGTTCGAGCACGCCATGATGCAGAATGTCCGAAATCCCATCATCATTGACCAAAATTACTGCCCCCATGAGAAAAGATGCCCTGGCCAGAATTCTGGAGTGAAGATTAGTCAGGTGAACTATATGGATGTCCAAGGGTCATCTGCATCACAAGTGGGGATAAACTTTGACTGTAGTGCCAGCAACCCATGCACCGGAATTGGACTGCAAGACATCAAGCTTACCTACGGAAACAAGCCAGCAAAATCGTTTTGTGAGCATGTCCATGGTACTGCCTCTGGTTCTGTTGTGCCGAGTTGCTTCTGACTCTTCGAGTTGGAATAGTATGTATAGATCGTATGTCAGAGTATGCATGTGTTGATGAAATGGGTGATGTAGGATTCGTAACTTTTCTTATCCTCCATGTCCAAGCGTTACTCTAGTAGACATGGAATGGAATAAATATTTAATGGATTGATATGTGTTTTTTATTATAATAATTTCGGCCTAAACTAACGTTCTCATTCATGGTCCCCAATTAAAAAAAAAANAAAAAAAGTAACTCTGCGTTGATACTACTGCTTAAGCAGTGGTATCAAC |
| ***EgPG6*** | **>CL1Contig8210_RC**  CCTACGGGAACTGTTTGTGATATTTTAAGGAAGGCAGAATTATTTCAAGAAAGAACTTCGATTCGAGTTAATTAAAGGAAAAAAAAAGAAAAAAANAAAAAAAAAAAGTAACTCTGCGTTGATACCACTGCTTAAGCAGTGGTATCAACGCAGAGTACGCGGGGGAACTGCCCTGCCGGTGCAAGGTCGTTAGTGTTCAGGAACTCGAAGAACATCTTGATCAGTGGACTGACGTCGATCAATAGCGAGAGGTTCCACATCGTGATCGATGGCTGCCAGGTGGTGACGGTGCACGGGGTCAGGATCACGGCCCCCGGCAACAGCCCCAACACCGATGGCATCCACGTCCAGTCCTCCACCGACGTCACGATCACCGGAGCCGGCATCAAGACGGGCGACGACTGCATCTCGATCGGGCCCGGCACGACGAACCTATGGATCGAGAAGGTCACTTGTGGACCTGGCCATGGCATAAGCATTGGGAGNTTGGGGAATGAGTA |
| ***EgPG7*** | **>CL1Contig694_RC**  TTCAACGAGTCTATAGCCTTGGCCGACAGGCCCGGGTAATCTTCGAAAATTTCATCGTGATGGAGTGACACTTAACACTAAAGCATTTCAAAATGCTATCTTTTATCTACAGTCATTTGCTGATAAGGGTGGGGCACAGCTCTTTGTACCAGCAGGAAGGTGGTTGACGGGAAGTTTTAATCTTATCAGCCACCTCACATTATGCTTAGACAAGGATGCAGTAATTATTGGATCCACAGATTCGTCAAGCTGGCCAGTTATCGATCCATTGCCTTCATATGGAAGAGGTAGAGAGCTACCTGGTGGGAGGCATAAAAGCCTCATTCACGGTTCCAATCTGACAGATGTGATAATAACAGGTGACAATGGGACTATTGATGGCCAAGGCAGCATTTGGTGGGATTGGTTCCATAATAAAACGTTGAACTTTACTCGCCCCCATTTGGTTGAGCTCATGTATTCAACCAAGGTGGTTATCTCAAATCTGACATTCATTAATTCCCCATTTTGGACCATTCATCCTGTATATTGCAGCAATGTCCTTGTCCGGAATGTCACAATTCTCGCCCCTCTTGATTCACCAAACACAGATGGAATTGATCCAGACTCAGCAAACAATGTCTGCATTGAAGATTGTTACATCAGCACTGGTGATGATCTAATTGCAGTCAAAAGCGGATGGGATGAATATGGCATTTCCTTTGCTCATCCAAGCTCCAATATTAGTATCCGCCGCATCACTGGAGAAACAAAATTAGGTGCTGGCATTGCGTTTGGAAGTGAGATGTCTGGTGGTGTATCAGAAGTTTGGGCAGAGGACATCCACCTCTTTAACTCAAAGCATGGTATCAGAATAAAAACATCTCCAGGGCGAGGTGGCTATATTCGAAATATCTACATCTCTGATGTGATCATGAAGAATGTGGACATAGCGATCAGGATCCAAGGTCAATATGGCGAGCATCCAGATGAAAAGTATGACCCAAATGC |
| ***EgPG8*** | **>CL1Contig4380**  AACGCAGAGTACNCGGGGGACTTCTCCTCTGTATCTCATTCTTCCTCCATAATTCCACCCCGTCTCTCTCTTAATCTTCTTCATCTATTCATTCTCTTTGCCGGCTCTTCTGGTTGGAGCTTCTCCTTGTGCCTTGTCCTGCCCCTGGGCTTAAGGGGGCTCATGAGGAGGCTAGTAGTTGTGCTATTAGTTTTAGCAGTTGCTAATGCTGTAGGAGTCGATGGAGCCAGTTATGAGAATTGCAAATATGAGAGGAGTTTGAACCAAAGGCCGCACAGTGTTTCTATAACGGATTTTGGTGCTGTCGGAGATGGGGTGACACTAAATACACTCGCCTTTCAGAATGCCATCTTCTATTTGCGGTCATTCGCCGACAAGGGCGGCGCTCAACTTTATGTTCCTAAAGGAAGGTGGCTGACTGGAAGTTTCAACCTCACCAGCCACCTCACCCTCTTTTTGGACAAGGATGCTGTTATAATTGGCACTCAGGAATCATCTCAGTGGCCTATTGTTGAGCCGCTACCTTCATATGGCCAAGGCCAGGATCTTCCAGGTGGAAGGCATCGTAGCTTGATAAATGGGCAAAACTTGACAGATGTTGATAACAGGTGATAATGGAACCATTGATGGTCAGGGTTCAGTTTGGTGGGGGTGGCTCCGTTCTCACACATTAAACTTTAGCCGTCCTCACCTTGTGGAACTTGTGAGTTCCAATGATATTGTGATTTCAAATCTGATATTTTTGAATTCCCCTGCTTGGAGCATTCATCCAGTATACTGTGGAAATGTGGAGGTCCGGAACATAACGATCCACACTTCATCCGATTCTTCATTTACAAGTGGTATAGTTCCAGATTCATGCTCAAATGTATGCATTGAGGATTGCAGCATTAGTGTCTCACATGATGCCATTTCTCTGAAAAGCGGTTGGGACAACTATGGAATCTCTTTTGGAAGACCTTCCTCCAACATTCACATCGACAATGTCCATCTGCAGACTTCTCTTGGTTCTGCCCTTGCTTTTGGCAGTGAGATGTCTGGTGGAATATCTGACATACATGTTCAGCACCTACATGTCCATGATTCTTTCACCGGTATAAAATTCAAGACTATCCGAGGCCGAGGTGGGTTCATGGAAAACATAGTCATATCGGATGTGGAGATGGAAAATGTTCAGGAGGCTTTTCAATTCACAGGACATTGTGGAAGCCACCCTGATGACCATTATGATCCTGATGCTCTTCCAGTCATCAAGCGGATTACCCTTAAGAATGTGGTTGGTACAAACATTTCAATTGCTGGTGTCCTCTCTGGAATTGAAGATGATCCCTTCACTGCTATCTGCCTTTCAAATGTCAACTTTTCTGTCACCTCAGATCCTTCTGCTTCTTGGGTTTGTTCATATGTCTCCGGGTTCTCCGAATCAGTCTTCCCTCAACCATGTGCCGATCTCCAAATCCCATACTCAAATTCTTCTCTCGTGTGCTTCTCGCTTCCAAACTACAGTGCTCTTGCAGCAGCCAAATGAGATCTTTTTGCTATCTTATCATTTGCTTCAGTCCCTCTGTACGATTGAGAGACTTGAGCTTCCATTACCATCTTGCCAATTCTTTTGCCAATAAATCCCAAAAAAGTCGGACCAAAGATTTCCTTTCTATCACCATTCAATTCATGCTTCTTGGGACTCAAGCAATTAGTTATCTTGCACCTTCAAACAGGAAAAAAAAAAGGGTGGGGGGGCCATCCGAGATGACAATGTGCTTGCCTGTACAGCTACATTTCTTCATTCAAACTGTAAGAAAAAGTTTTGGTAGTTGTTGGAATCCAGACATTGATTGACACTGTTGCATTTGCCATTAAACCATTAGAATTTGGTTGATATTTGTATATTCTATCCTGTGTGGACATGAAAATGAATTAAATTATCTGGGAATGGGATAGGAGTGGTTTGTGTATGCCACATGGCTTCATGTTATTAAAATAAATGGGTGGTTGGCTGTTAAAGTTATAAAACAGCTTGATCTAGAAAAAAAAAAAAAAAAAAAAAANAAAAAAAGTACTCTAGCGTTGATACCA |
| ***EgPG9*** | **>CL8640Contig1**  TCATGGCTCCAGATGAGTCCGACGAGGCCGGTGGGGGTCGCAACCCCTCCGGCCTATGGAGACGCCGGTGCCGCGCCGGTGGAACACCCGAGCAGCAGCTGCTCGGGTTTCTACCGGCCCGGCGGGGGAGGAGGGTGGTTTGCTCGATAGAGGAATTTGGCGGCGTGGGGGACGGGACGACGTCGAACACCGACGCGTTTCGAAGGGCGGTGGAGCACCTGAAGGCCTTCGCCGATGAGGGCGGGTCGCAGCTGACCGTGCCTCGGGGAAGGTGGCTCACCGGCAGCTTCAACCTGACGAGCAACTTCACCCTCTACTTGGAGGAGGGTGCAATTATTTTGGGATCACAGGATCCAAAAGAGTGGCCTCTGATAGAGCCATTGCCAATCCTATGGGCGTGGAAGAGAGAGGTTAGGAGCACGCTACATTAGCCTCATCCATGGAGATGGCCTCAGTGATGTTGTCATCACGGGACAGAATGGGAGCATTGATGGACAAGGGAGGATGTGGTGGGAGCTGTGGTGGAATAGAACTCTGAAACACACTAGGGGACACCTTCTTGAGCTAATGAATTCACACAACATCCTCATATCCAACCTCACCTTCATCAACTCTCCATTTTGGACAATCCATCCAGTCTATTGCAGCAACGTGGTGGTAAAAATGTAACCATACTGGCACCACTTAATGCTCCAAATACTGATGGCATTGATCCAGACTCGAGCTCAAATGTGTGCATTGAAGACTGTTACATTGAGAGCGGGGATGACCTGGTTGCCATAAGAGTGGCTGGGACCAGTATGGGATCGCCATGGCCTATCCTAGCTCAAACNTTGTTGTTCGCAGGGTCTCCGGCACAACTCCGA |
| ***EgPG10*** | **>CL4959Contig1**  GTGCCGCCCCGCCTCGTGGGCTTCATCGACTCCAAGGACGTTCGGATCTGGGACATCACGCTCAACCAGCCCGCCTACTGGTGTTTACATCTAGTCAGGTGCGATAACACATTGATTCACGATATATCCATTTATGGAGATTTTGATTCCCCCAACAATGATGGCATCGACATCGAGGATTCAAACAACACAGTCATCACAAGGTGTCATATTGACACTGGAGATGATGCAATATGTCCAAAATCTTCGACAGGGCCTGTATATAACTTAACAGCAACAGACTGTTGGATTCGCACCAAATCTAGTGCAATTAAGCTTGGAAGCGCTAGCTGGTTTGATTTCAGAAGGTTCTTCTTCAACAATATTGACATTGTGGATTCACATAGAGGGCTTGGGATGCAGATACGAGATGGAGGAAATGTGAATGACATTGTCTTCTCTAACATCAGAATAAGCACAAGATACTATGATCCTTCGTGGTGGGGAAGAGCAGAGCCTATTTACATCACAACCTGCCCGAGGGATTCAAATTCAAAAGCTGGGTCTATCTCCAACGTTCTCTTTGTTAACATATCAGCAACTTCGGAAAATGGAGTGTTCCTATCGGGTTCCGGTGGTGGACTTCTTAGAAATCTAAGATTCGAAAATGTCCAATTAACTTATAGAAGAACAACCCGCTATCCAGGTGGGTTGTATGATTACAGGCCTGGATGTCAGGGCCTGGTTCCTCACAGAATGGGTGGAATCATGATGGAGCATGTTTCAGGTCTGGAGATAGAGAATGTGAAGATGAGGTGGATTAAGAGCAACTCAAGAGGCTGGAACAACCCTCTTGAGTTCACACCTTCCACTGTAAACAAGCTCTCTTTCCATGAATGGCTGTCAGATGTCTCTTAGCCAAAAGAAAAAAAGAAAAGATCAAAGGATTTCAATTTGAGTGAAGGCTCCCAGGTCATTCTTATGCAAAGTCAGAATAGATACTATGTCTCTATTTATTGTTATCATTTGCTCATGATTAAT |
| ***EgPG11*** | **>M01000011018:1,1896**  ccgtgccaacctgctgctttctccgtccttcttcacaaatggcttggcggaggcacagtagcatctcactccaaaccttggcgttatggatttcactaatggcggtattcatcggaacggcggaatgcgcccggcggagcatccacctccccggcggcagccgcgatggcggggtgatgggggcgttccagtacgcggcgacgagctgccgggccaacacggcgtcgctgaaggacttcggcggggttggcgacggcaagacctccaacacgaaggccttccaggcggcggtggcgcacctggcccagttcgccggcgacggcggcggtggtatgctgtacgtgccggcggggcggtggctgacgggccccttcaaccttaccagctccttcaccctcttcctccaccgcgacgccgtcatcctcgccacccaggatattagtgaatggcctgtcatcgatcctctgccctcgtatggaagaggaagagatcatgctggtggaagatacagcagcctcattggaggatcaaacctcactgatgtaattatcacaggggacaatggaacaattgatgggcagggggccttctggtggcagcaattccatggaggcaagcttaaatacacacgcggatatctcattgaactattacactccgaccagatatttatatccaatcttacactactcaactctcctgcatggaacgtccatccggtctacagcagcaacattataatttcgggcatcactatactcgcaccactccattctccaaacacagatgggataaacccagactcctgcacccatgttcgaattgaggactgctacatagtctctggtgatgactgcgttgccatcaagagcggctgggatgagtatggcattgctttcggaatgccgagccagcacatcattatccgaaggctaacctgcatctccccgaccagtgccgtcatcgctctagggagcgagatgtcaggtggaattcaagatgtgagggctgaggatatcaccgccatcgattccgagtccggtgtccgaatcaagactgctattgggaggggagcatatgtgaaggatatctttgtcagggggatgaatctcaacaccatgaagtgggtgttctggatgactggagcctacaagtcccaccctcgataacaagtatgatcccaatgccattccggttgtcaaagggatcagttacagtgatgttgttgccacagatgtaacacaagcagcaaggttggaggggatttcaaacgcaccatttacaggcatttgcatttctaatgtcacagtccatttagcagcaaaggcaaagaagcagtcatggacctgtactgatgttgagggtgtgtctagtggagtgagtcctacaccttgtgcttcactaccagatcggggtgccggtgctgagccatgcccgttcccgaccgacagactacctattgatgatgtggtgtttgaggagtgctcatacatgagctaataatctccgaggttgcattcgcatatggttcgttcatatagtgtttatcaagtcaatcctcatgtacaatggtggctggtggcagagcttttgtgtgcttctagcttcttgatgcttaaggtttgctataaaaatgttaaagccttgaaggttatggagtaatccattcctcctttgtgtgctagatctatgttcttacatttcaaagttctataatatttccacataacttgattacagaggactgtatactaaaaatgcatgatgccatgatgttattacttggacagggtcttgccttcattttgatagaaatggtacatgaaaattgaacaaagaaagactgcaattgacaacaagattttagtggagcaataaaatgttgatacaaatatt |
| ***EgPG12*** | **>M01000058719:1,1373**  tgaagggatacttgaagggaacaacagatttgagccagtatgtcacaggtgattgggtggatatgttgatattgaccggaggagggaccttcgatggccaaggagctgtatcatggccctataacaagtgccctacaaataaacattgcaaagtcctccccacttcggtcaagttcgttgtcacatcaaacactctggtacagaacatcaaatcaagtttttccacatagctctggttggctgcaagaacttctggggcaaaaatatccagatcaccgccccttcaaacagccccaacaccgacggaattcacattgaacggagcagaggcgtaactatatataactcggtgatcggaactggtgatgattgtatctccattggacatggtaactcacaagtattactgagtggcatcagttgtggaccagggcatgggatcaggcaagtcctcttgaagttctcctgctacaatattgttatgttctgattgaaatttttgatccaatccaaatgagaacagatccttggattaatcctgggtttagactgatgtccgatgcacttctatttgaccctcaccagaccccgcaatgcattgggagtttaggaagatatcataatgaaggggacgtccgaggactcgtcatcaaagatagcacccttgccgggacttcaaacggtgtaaggatcaagacatgggagaactctccaggaaccagtaaggctgttaacatgacctttgagaacattgtcatgaacagtgttgcaaatcccatcatcattgaccagatgtactgcccctacagctcttgtgcatcagatgcaccatctggggtgattctgagtgacatcttcttccggaacataagagggacgtcgacgactccggtggcggtgaccctcaggtgcagcagaggagtgccatgcaagaacgtcaatctccaagacgtcaacctcaagtacgttggccagcttccggccactgcctcgtgcatgaacgtcaaagcaagcttcagcgggacccaaatccccccaccttgccgctaggcaaaccgacccaaaacttagctaattggcccccttggtgctcgtggaactcattgaagccattcttcctggcctccatctaaggctgggttacattctttcctgtgggttgtaaccatcttaccatcaagcatgagtaatttgtttttggattatatttacaggctgtggaaatccggggactgagtaatttgtttttggatggattatattgagaggtggctctggttgaaccgccattctttctgtggttatctttctttctttgtattttttgggaaccaatgttgttatctttgtttagttgttgggattttgatttg |
| ***EgPG13*** | **>M01000001841:1,1933**  ctaaacattcttacgctttttttctgtttccatcttcacggcttggcggagctcctctgcttctccctgggccaagaaaaataaaaactaaacaacttcagatggcgtcaagaccaccgatggcgctggtactggcagcgttccttctcttggtggaatggggctcggcggcggcggcggcggcggcggcaggggaggagacgtgctcggggatagtgccgatgaaggagcggggaacggcaatgtcggtggaggatttcggcggggtgggggacggccggacgctcaacacggcggctttcgagaaggccgtggcggagatcgaacggcgaggggtggccggtggcaccctctacgtcccgccgggggtctggctcacgggggccttcaatctcaccagccatatgacccttttcctcgccaggggcgccgtcatcaaggccacccaggatacatcgagctggcctttgatcgatgctttgccatcatatgggagaggtcgggagctacctggtgggagatatatgagtctgattcatggaaatggaattcatgatgtgataataacaggtgagaatggaacaattgatgggcaaggcgaggtctggtggaatatgtggaggcaaagaaccctacctttcacaagaccaaatatattggagctcatgcattctaaagatattattatttctaatgtggtcttccaaaactcaccattttggaacattcaccctgtttattgcagtaatgtggtagtaagaaatgtgaccgtgttggctccatatgactctcctaacacggatggaatcgatccagattccagcttaaatgtctgcatagaggactgctatatttcaacaggggatgatctagtagctgtgaaaagtgggtgggatgagtatggcatggcctatgcccgccccagctctggcatcaccattcggcggttgaccggctcctctccttttgccggaatcgcagttggaagcgaaacctccggtggggtggagaatgtcttagtggagagcatacatatctataacactggtattgggatacatataaaaaccaattctggtaggggtgggtatataagaaacataaccgtctccggtgtgaatctgagcaatgttcgcaaggctctgagaattgcaggtgatgttggagatcatcctgacgacatgtataatccaaatgctcttccaattgtcaatggtttgaccatcaaggatgtgtggggtgcggatatccagcagcctgggtcgatagatggcatcaagaactcacccttcacaggggtatgtctttctaatgtcaagcttaatggtgatggaacacgggaagtgccatggaagtgtgcggatgtgagtggtggtgccctcggggtgcagccatcaccatgcacggagttgacaagtatttctgggttgagcttttgtacgaatgcaatctgatgttctatcatttgttggatgccgttgtcactaagaaaatgcatgtctttgcctatttggctgatagaaaaaaaatttgagcataaaatttggtgctattataaagatatgcagcttgtatatgaaagaatctatatctcatgattttcaaagcatgaaaatatttgaagttatcgaccaagtgccggaggtattgccgatataagtatcaaccaggcagaagataagctctgtttctgtgcaactaacaaaaatgcgggttgatttttattgaactagagtggctatcatagtacagttgcagtgttatgctggaaagagatctaagatatggcatactacaatttgcagtattatgctatctagttaacaagcagcatttgcaaacgggcttaaattgttatctattgtaatttcattggatgcatatacagttattctagagtatggtttaatatttgatggtccttgactggatg |
| ***EgPG16*** | **>M01000013617_RC:1,1490**  ccggacaaggcggcgccgaggctccgtccgtttgcgtacaatatcacggactttggtggggttggcgatggagtgacgctgaatactgaggcatttgagcgcgcggtggcggcgatcgcgaggcatagggggaggggcggcgggcagctcaatgtgccgcctggagtctggttgacggcgccgttcaatcttacgagtcatatgactctatttcttgcagaaggtgccgagattcgtggaattgaggatgagagatattggccattaatgcctccagttccatcatatggatttgggagggaacataagggacctcgatatggaagtctgatccatggccagaatctgaaggatttggttataacaggacacaatggaaccataaatggacaaggtcaagcatggtgggcaaaatatcgcaaaaggcttctcaacaacactagaggacctcttgtgcagatcatgtggtccaaagacatagttatttccaacataactctgcgcgattctcctttttggacactccatcctttcgattgcaaaaatgttactgttacaaatgttaccatcttggctcctgttactggagctccaaacacggatgggatagatccggattcatgtgaggatgtggtgatagagaactgttacatatgcgtaggtgatgatggagtggccataaagagtggttgggatcagtatggaattgcgtatgggcgcccatctactaacattttaatccgcaatctcactgttcgctctgtggtgagtgctggagtatcaataggcagtgagatgtctggaggagtttcaaatgttactgtggaagaccttaatatctgggagtccaggcggggcattagaataaagactgcccctgggagaggaggctacattcgcaacatatcctaccacaatgtgacccttgataatgttcgtgttggcattgtgatcaagaccgactacaacgagcaccctgatgaaggcttcgatcccaaggctgttcccctctttgagaacatatccttcagtgggattcatggccagggagtccgtgtcccagttcggatccatggcagtgaggacatccctgttaagggtgtcagcttccgtgacatgtctgtgggtataacctacaagaagaagcacatattccagtgttcctttgttcaaggacgtgtgattgggtccatcttccctgcaccatgtcagaatctcgacctttatgatgagcaggggcagctggtgaggcaatcagcatcacagaacagcaccgacattgactatgatatttgaaaattttgttccctctccattgtccagatgggaggagtgccgggttattccgtggcagtgatggttctgtcaagcatctgttcccaaggtttcaacccccctggcttgtacatacagagctttcaatttttgatttttttttttttttttggtaattatctggtgatatgcctcaccacttcatgatcttgcag |
| ***EgPG17*** | **>M01000033226:1,1913**  gtctctctgtgggacggttgcagtctttctagctaataatggcgttgctcggaagcaatccctcaagaaacctctacgtactaaagttggtgattttatggatggtggtggcgggacttatgagggcggcggagtgcacgaggctaaggcaccacggccaccaccgccggaggcccggcggaggcttgcccgcgggggcggcggcggcgggtgccttcggctgccgagcctacaaggcgagcctgacggatttcggggcggtgggtgacgggaccacctccaacacccacgccttcgccgcggccgtcgccaacctcagcctctacgccgcccacggcggggcgatgctcttcgtgccccccggcaaatggctcactggtcctttcaatctcaccagccacttcaccctcttcctccaccgcgacgccgaaatcctggcctcccaggacattgatgaatggccaattattgatcccttgccctcttatgggagaggaagggacttacctggtggaagatacagcaacttcatcatgggatctaacctcactgatgtgatcatcacaggggataatggaacaatcaatgggcaggggaaagtctggtgggataagtttcatgataacaagctggagtacactcgtggttacttcgttgaaataatgtactcagaccacatccttatttccaaccttatattcaaagatgctcctgcatggaaccttcatccggtttacagcagcaatgttcttgtgttaaatgtgacaattcttgcaccggttacctctccaaacaccgatggaattgatccagactccagctccaacgtcctcatcgaggactgcttcatagtctctggtgatgattgcatcgccatcaaaagtggttgggatgagtatggaattgccttcaacatgccaacccgacatgtaattattagaaggctcacctgcatttcccccaccagtgccaccattgcccttggaagcgagatgtctggtggtatcgaagacgtcagggctgaagatatcacggccatcaacacagaatctggggtcagaatcaagacggccattggtaggggaggatatgttaaggatatttttgtgagaagaatgaccttgaacaccatgaagtatgttttttggatggctggaaattatgggcagcaccctgatgacaagtatgaccccaacgcaatcccaataatccacaatatcaattacagagatgtggttgccgaggatgtcaccatagctggaaagttggagggacttcctaaggcacccttcactgggatatgcttgtctaatgtgactgtggatgttgtgaagtcgaagaagctgaagtggaactgcactgatgttgagggggtctcaagtgctgtgatgcctactccttgcgactcactccgagatctaggagctgatgatgctccatgccctttcccaacaggttctttacccatagaaactgttgcattccaggaatgtcaatacggaagagttaaactgtgagtgttggtgtgagctttgttttattttccttggagtcattcctcatgaccgatgtctgcaaggagaagagctcttatgtgcttctgtctcttgataattggaggttcgatgaggaaaactagtgttgcaataagtcatttgtttctcctaggtattggagttttttcctttttaaagtatattatgtcatgtgagtgcatgtatgtgaagctggacctaaatttgtgaccaacaagtggtagtttaatggctacaggggctactgcaagtgattctaaacctttttggattgctatgctagcttcatcaggtcagcctaaaccttttacttcattatgtaaattaaactttatcatctgaaagtttatatatatatatctggaatttgtatcaaaa |
| ***EgPG18*** | **>M01000001611:1,1662**  aacaacagatttgagccagtatgtcacaggtgattgggtggatatgttgatattgaccggaggagggaccttcgatggccaaggagctgtatcatggccctataacaagtgccctacaaataaacattgcaaagtcctccccactgtaaactctcaagcctcatttatcctttatgctctctacggtagatctaatacttgcaactcgagtatatatagttaccacgtacacgtcctttctaatttgacaattttgcacaaaagtagaaataatgtatgaaatcaagccaatcacatactggtgcattttagagctttaaaactagctcactgcataaggcaattagtatttcccagctagtagctacttcaccaaaagtttcacagtggaataatggcagtgccagattttttttgtccgcatcaaggaagaatgaataactctatataggcttagattaatgaatattctagactagagtagtactttcatgttgctacccaaactgcagtagcaaaatttgtccaaccgttgtcttttagatgtaacacttgccctctgcattttagggtgaagtatttttctaagacaagcattgtagcatgaagttatagcagctagctatcaatactcataattgccagcatcattaagatcacagatgttagtaaaaactgtagaagctctatagtccaaatttgctattcagcttatcatctaaacatgcttaattgtcattttggtgattatcttgagcatttcagtcggtcaagttcgttgtcacatcaaacactctggtacagaacatcaaatcaagtttttccacatagctctggttggctgcaagaacttctggggcaaaaatatccagatcaccgccccttcaaacagccccaacaccgacggaattcacattgaacggagcagaggcgtaactatatataactcggtgatcggaactggtgatgattgtatctccattggacatggtaactcacaagtattactgagtggcatcagttgtggaccagggcatgggatcaggcaagtcctcttgaagttctcctgctacaatattgttatgttctgattgaaatttttgatccaatccaaatgagaacagatccttggattaatcctgggtttagactgatgtccgatgcacttctatttgaccctcaccagaccccgcaatgcattgggagtttaggaagatatcataatgaaggggacgtccgaggactcgtcatcaaagatagcacccttgccgggacttcaaacggtgtaaggatcaagacatgggagaactctccaggaaccagtaaggctgttaacatgacctttgagaacattgtcatgaacagtgttgcaaatcccatcatcattgaccagatgtactgcccctacagctcttgtgcatcagatgtaagcaagctaacacaatcatgtcatgcaaaaatatgaggggctcatataatattttttccgcatatagtgcttagagcttgataatgcatgcatttaaacaacaaaacttggccagggcttggaggagatggtccgctgtggtttctcctgtgaataggctctacttatctttccctgctacagaagaaggcattacttagtttgacaataaaattgtgcaaagtactaaatcc |
| ***EgPG19*** | **>M01000015126:1,1043**  gcagttgcatcctatatctcaggaagtgcccagctggattgctttgtttttagttgtctcaagtaaaaagacaactgataatatcatttttttaatcttaatttttgtatggaatcagcatagggagcctcgggaaggacaatgcgactgccatagttacagcaatcgtcttggacacagcaacacttacaggcactaccaatggccttagaatcaaaacctggcaaggaggatcaggctatgttaaatcagtgcgctttgaaaatgtgaagatggatgatgttgagaacccaatcatcatcgaccaattttactgtgattctccaaccagttgcaagaaccagacatcggcagtgaagataagccaagtcctctacaggaacataagtgggacctcaaggactcctaatgccatgaaatttgcttgcagtgacacagtaccatgcagcaacattgtgttgaacaacataaatctagagagagaaaatggcattgtagagaccttctgcaactgtgccatgggatttgactatgggtttgtgcggccagcggcagattgccttcgcaatgctaaaaattcccgctgtgatgggacaaagaatgacaaccaaaaccaaacttatgatcccgtgcatacagagctatgatctctctatatcatatcaaggtatcatattacagaccacgataggtggagaccaacaaaaaaccgtacttacaagtacgcctacatgttactgagttctggcatattgaatagaaagatttagtggaagaatttgagctattgaagcagacacaagctagcgactttgacgcatatatcttaggcaatcaaaaatggtatagaagaaagaaatcgatgtacttgaactctaaagatatcctgtatgacttttaaggaacaaccgtatttgtccatgcttcataaatggaaacagaaatcacaaggacactctttctggactattggatccaggggatgtaaaacccataccactaccatgtcaattgtagaagcatttatgcagcaattattgttc |
| ***EgPG20*** | **>M01000028952_RC:1,1051**  ttttcggtattgtattttagttgcatgacttttgtctcatctgtcttttctatagttattttctcgcaatatgtgcttggtagattctttttttttttttgttttttattctaattgctttttttttagttatgattcgatcatgatccattgttttttactctttcttttcttctcccttcaactcttctctcattccttttttttttgaataataaaatttaaataactaacgtttaagtttttcttttgcttgattctttttggaagtattttgttctgcatgccgggaggccaatttaaattgaagctgaaccttccttcatgatccaacataacgagaagataatgtgatctcagccatctattgaatggacttcagaaactgatcatcacatagaaatctatcttccaaatgttacggttgttgtcatttatttttgttttaaagacataatggcttttttaataaactacaatgcttcaaaactttttgtttggagataatatactgtccttaattctaaagtgagaaacattgcatcaaaggtagtcttagaacatcaaaagcaaggtagcaatgtttaaatctaggatttgttcttgctgtgctaattgtttccttgttggtgatgggtaagctggcttttaatcaattagctaaggcattcatggccgcatggaaggcagcatgcgcagcagttggtgtggtcaagcttcagatacctgcaggaacttatcttattggccctactaagtttgctggtccttgcaaaaatgttcactcccttacagtgaatatgaagggatacttgaagggaacaacagatttgagccagtatgtcacaggtgattgggtggatatgttgatattgaccggaggaggggaccttcgatggccaaggagctgtatcatggccctataacaagtgccctaccaaataaacaattgcaaagtacctccccaccttcggttcaagttacgttgtcacatcaaacactctggtacagaacaatcaaatacaagtttttacctaccatagctctggtt |
| ***EgPG21*** | **>M01000034881:1,1267**  gcgtttcgaagggcggtggagcacctgaaggccttcgccgatgagggcgggtcgcagctgaccgtgcctcggggaaggtggctcaccggcagcttcaacctgacgagcaacttcaccctctacttggaggagggtgcaattattttgggatcacaggatccaaaagagtggcctctgatagagccattgccatcctatgggcgtggaagagagaggttaggagcacgctacattagcctcatccatggagatggcctcagtgatgttgtcatcacggactcgagctcaaatgtgtgcattgaagactgttacattgagagcggggatgacctggttgccataaagagtggctgggaccagtatgggatcgccatggcctatcctagctcaaacattgttgttcgcagggtctccggcacaactccgacttgctcagggattggatttggaagtgaaatgtctggtggaatatccaacgtcttggtggaagacctgcatgtctggaattcagcagcagcagtgagactgaagactgacagagggaggggaggatacatttccaacatcacaattgctaatgtgaccatggaaagggttaagattcccataagatttagcagaggctctaatgatcatgccgatgaaggttatgacccaaaggcccttcctagaataaatggtgtttacattaaaaatagtagccggcatcgacgtagggaaggcgccggttctggaaggcattgagggaacaatatatgaaaagatatgcttcagaaatgttagcttagggaggttaagccctaaggccagatggcattgtgagtttgttgctggagaagcttatgatgtgttcccaacgccatgtcaacagcttaagaacaatggttcttcatcttggtgcagatactcctaggccatatagacaggaatcaaggttttcagcctcaactctgcctcaaagataatgaggtggaatgatccataacaatcttgaagtagtagtccaaatggtatacatgagggtgtggtatcttggagatgttagctttgggattagttcgtttgtattttcttttatttggtaggttcagatataaactagaaagtagcatataggattaacaagatacatattccatttatttgtacttattaaagagagtgacgtaggtctttgtgattcttttagtcaaggaagtttattctataaattttttttttatcaatagtagaggaagactgaaagttcctttttctttaaaagaag |
| ***EgPG22*** | **>M01000018577:1,1075**  Agtcagagcaatcgacttagtaatagagggcgagaagtatgtcgtgcttctccctgcttcttatgctgatgttagttgtactgatgatgggggccgtgtccaaaggtgatgacaattttgccccatctgaggatatcgggccctggacggtcggtacggacggcgaggaagagcaagaaggagacgagtatgatgaattggactccctaccggcatggggaagcgagcgggttgggaggatccctgtcaatgtcgacagctttggtgctgtaggggatggagttgcagacgatacccaggcatttctagaggcttgggaaacggcgtgctccataaagaatgcagttttcctagtgcccgagcggcgccgttacaaagtcaacgcaaccaggctacgagggccgtgcgaaaggaagctactcatccaggtgagcgggacaattgtagcccctgatgagccaaacgactgggacccgaagaacccaaggacatggctatctttctctatgctgaagggcgtcaggatccagggcggtgggattatcgacggctcaggcagcaaatggtgggcgtcatcctgcaaaatcaacaggaccagtccatgcagaggagctccaacggcactgaccatagattcaagctcaaaggtaagggtgaaggacctcaccatccagaatgcacagcagatgcacttcaccatctcccgatctgatgccatacgagtgtctgggttgcgtgtgagagctccacaagacagccctaacacagatggcattcatatcagtgaatccaccaatgttgccatccagaactgccacattggaacaggggacgactgtatctccattgtgaatgctagctcaattatcaagatgaaaaacattgtgtgtgggccagggcatggaatcagcatagggagcctcgggaaggacaatgcgactgccatagttacagcaatcgtcttggacacagcaacacttacaggcactaccaatggccttagaatcaaaacctggcaaggaggatcaggctatgttaaaatcaagtgcgctttgaaaatgtgaagatggatgat |
| ***EgPG24*** | **>M01000015281_RC:1,623**  aaaaaagatccttgttaccccacctactccttataccaatcaaggataagtacacttcctatccagtaggcttgatctattttttatgttcagtgtgttctctgtatgtatgtatgatgaacagaagcattcatggagaagtattttgccaggggattctaagagttgctctaaagttttttttctttttcctccttggatttcatctgatctgaggtctcactacttagtttctgaggcatggttttgtgagccaggtctatggatgatgggtgaatagggttgtttttgaaagctttatgttttcattttttaattttttattgaatggtgcaggatacatcgagctggcctttgatcgatgctttgccatcatatgggagaggtcgggagctacctggtgggagatatatgagtctgattcatggaaatggaattcatgatgtgataataacaggtgagaatggaacaattgatgggcaaggcgaggtctggtggaatatgtggaggcaaagaaccctacctttcacgagaccaaatatattggagctcatgcattctaaagatattattatttctaatgtggtcttccaaaactcaccattttggaacattcaccctgt |
| ***EgPG25*** | **>M01000031930_RC:1,710**  caaactatgagtacaactttggaaagccttattaacctaacttcaaagatgtcaaaactggtatttttatttcaaagattgttgttacataggcaagcctttctgagtaagctggaaattaattagctttgcagataatgggaactcatagtttttcttgtattttaattgcattgcctttattttctctgaataataattttcgataataactggccatcttatctttaatcagaacctgatcatgaatttgagtgcattagtatatgcacatataatgactgtaatatgtagaatatcttgtatgtttatatatgtacgtaggtatgtgtatatgtacgtatatatgtatggctgtatgcattcatgttatttcgttctttaatttatctcattgcatactagtatgattctatgaactaattaatatgggcattatccttaaaaacattacgttaaatttcttttgtcccacttcttgttcccaggtgagaatggaacaattgatgggcaaggcgaggtctggtggaatatgtggaggcaaagaaccctacctttcccgagaccaaatatattggagctcatgcattctaaagatattattatttctaatgtggtcttccaaaactcaccattttggaacattcaccctgtttattgcagtaatgtggtagtaagaaatgtgaccgtgttggctccatatgactctc |
| ***EgPG26*** | **>M01000039313:1,541**  Ggagaatgttaaaaactgcatcattatagaccagtactattgcctaagcaagcagtgtatgaatcaaacatcggcggtctacgtaaaggatgtctcatacatgaacataaaggggacgtatgatgtcaggagcccaccaatacactttgcttgcagtgatgccgtgccatgcaccaacatcaccatgtctgatgtggagctgctcccatttgatggagagttggtggatgacccttttgttggaatgcctatgggagcatggagacactaacaattccacccatctcttgcttgcaggaaggggagcctcagtccatcaaagagaactcatacagtctcgggctgtgctttgtggtggcattagaagagatatatctgtcaatcctgtaagttcacgagctcttgcaaagaagaggaccttaacctcgatccatggacccacttccctttcttccatgtcacttgttttctgcatcttggttactccacccgctttattagcagcacaagtgtcttcggattcttccgccatggacgtaac |
| ***EgPG27*** | **>M01000041301_RC:1,603**  tgataaggcactctgataatggtctaaggatcaagacatggcagggtggtatggggaccgtatcgagtattagcttcgacaccgtatacctggagaacgtaaggaattgtatcatcatagaccaatactattgcttggacaagaagtgtaggaatcaaacatcggcggtctacgtatccgatgtctcatacactaacatcaaagggacatacgatgtaaggagtgcccccatacactttgcatgcagtgacaccgtgccatgcaccaatattaccatgtcggaggtcgagctactaccttatgaaggggagcttgtggatgatcccttctgttggaatgcttatggggtcacacagaccctcacaatccctcctatcctttttgtttgcaggactgggcagcctcagaatctccaggacagctccaattataattgctaacacaacataacatgctttggccatagcaaatagtagccacatatagaacatagaataaagtccaagacccgtacagctgatgaacaatttaatttttcttcgttgttctggccattgtggggcttgcatggtgtttgtgaaccttttctatatgtatttgctt |
| ***EgPG28*** | **>M01000041909:1,534**  Cctgctacaatattgttatgttctgattgaaatttttgatccaatccaaaatgagaacagatccttggattaatcctgggtttagactgatgtccgatgcacttctatttgaccctcaccagaccccgcaatggtaagagcctcgtgtactagggtacctccctttttttggtttccgactttttatgggttagatgtggcccaagtaaggtctagaactgctaaatgagctaaattgtgctgccacagctgctgctaatattgctgatgcagtttcattttatatggtcattgcagcattgggagtttaggaagatatcataatgaaggggacgtccgaggactcgtcatcaaagatagcacccttgccgggacttcaaacggtgtaaggatcaagacatgggagaactctccaggaaccagtaaggctgttaacatgacctttgagaacattgtcatgaacagtgttgcaaatcccatcatcattgaccagatgtactgcccctanagctcttgtgcatcagatgcaccatc |
| ***EgPG29*** | **>M01000051458:1,630**  aagggtagtttagttctcataatcttggtgatattagtttaatttatcgtgtagatgaaactcataggcaagtttttggtgcacacaataatatgagaagccctctgcctgtgttccctgattcctctatcctgaattaggtgggactggttcttaggtctctgacagagcacaaagtcatcatcaatgaataaactttaccagtgaactctaacaagaacattctgatgcaatcctattatgacattgccctactttagatttggtgctgtttcctatcttaagccagaagaacataaactaatatgagcagtgagtttagctaaatctttcctaacgttcaccctaagaaaaataaattaaaatcaacacacaaaaacaatagcaatgagtgtatgtgattgaatgcaggcaccatctggggtgattctgagtgacatcttcttccggaacataagagggacgtcgacgactccggtggcggtgaccctcaggtgcagcagaggagtgccatgcaagaacgtcaatctccaagacgtcaacctcaagtacgttggccagcttccggccactgcctcgtgcatgaacgtcaaagcaagcttcagcggggaccccaaaatccccccncct |
| ***EgPG30*** | **>M01000063855_RC:1,693**  agtttactctgctaagaaaaagagcaaacaaagctacaattctgcttcaaagttgctaacgctactttctccagttcagcatggcgatccttcgatcgctgttctgtgtctggatgttggtcacattgctgtcattgggtccccagagctcactcggaagatctcatttccataagaagcaaaagagtactccgaaaaacggtaaaggccatagctctgtgtccccctgtaaattccccagcaaacgcaccaggtactaccactaccgatccctgcaattcaagctcggatccatgtatcttcgatgtgagatccttcggtgcggtcggtgatggttccaccgatgacaccgaagcattccggtcggcatggaaggaagcctgctccgtcgagtcaggtacgctgctcgtgccgtcggatggcgtcttcatgatcacttccacaatcttctctggcccttgccaaccggggcttgtgtttcaagttgatggagttttgatgccacccaacggccccgactgctggccagagtccgacagcaagctccagtggctcgtgttttatcgactcgatgggatgactttgagaggagaaggaacaattgaagggaatggagaagagtggtggaacctcccctgcaaaccccacagagggccaaatggatccacattgcccggaccttgcagcagtcct |
| ***EgPG32*** | **>M01000031649_RC:1,535**  gtaccgagaatagttgtatagggcctacaatagactgaaataggtccctcactgccaaggaggtgagaaagaaagaaaggaggaccaagagaatcagcctcacagttccacttgggccatcttttagcttcccctcctttaccaagtagagatggatctgctacactcccttgagattgaaacgaggcataaaacatcatcaaagattgtttctgttcctcacctttccttgtatagatccactgtataacacaacacatactgcagtggtcaaacaagtgtgaaaatcccatatgtttgctgacattagagttttggcttgtgtcatacaggtgatgactgcatctccatccaaacaggatgctcaaatgtatacatacacaatgtgaactgtggaccaggccatgggattagcataggcggacttgggaaggacaacaccaaagcatgcgtctccaatataacagtaagggatgtcaacatgcataacacgatgactggtgtccgaatcaagacctggcaggtaacaaacttc |
| ***EgPG34*** | **>M01000044771_RC:1,534**  taaattttccagtacaagtcaggcaataaagtaggccttggctaatagtcggtcgaagctgcacaacatggctagaccagcaaagtttctgtcgctgaagaaaataaaaacaatacttataggcaatgctgtcgaggtgacaatccactgccaccatcttcaccatcaccgccatttttttgtgtaggcactgaccatagattcaagctcaaaggtagggtgaaggacctcaccatccagaatgcacagcagatgcacttcaccatctcccgatctgatgccatacgagtgtctgggttgcgtgtgagagctccacaagacagccctaacacagatggcattcatatcagtgaatccaccaatgttgccatccagaactgccacattggaacaggtaccttgacatgcttgtacattgatctaccattctgatataccatacatgaaagattacctgaaggaaacgagaagaataataacaataacagtgattattcattgacttcactatgactgaaactgcaattaacttcc |
| ***EgPG35*** | **>M01000046001:1,808**  Agcataggaggactcgggaaaggcaatagccctagcctgtgtctccaatgtcactgttgatacgcatcaatgttcaaaatgctctgtctggagtaaggatcaaaacatggcaggtaaatactaagaagacatgtaaaaaccttgatttagatgtactgtaatactaaatatgtgtccaccaaaaaagccatgtaatcttaccttgtgatttaagagatgctacaaaaatagttgatttataactcctaaactttcatcacagggaggtctaggatctgtcaggaatgtcatattttccgatgttcgagtctccaatgttgagatcccagttgtgattgatcagtattactgcaacaagaaggcatgcaagaacaagactgatgcagtggctgtttcaggagtcatgtacaaaaggataactgggacatattcataccaaccaatgcatcttgcttgcagtgacagcaatccatgcacgagcatcaaattgactgatatccggctatcaccagttaatgcatctcaatttcagcaggacgccttctgctggaagtcatatggagagtcacaaggtcctcttgagcctttaagcattggttgcttgcaaaggaccagtaggtccatcaagcccctaataaagtcatccaatcacacttgctaggaagtatggctattcaatttctagggctactatattgttatcatggcttcccctttctgaactttggacagtaagaaggtgatggtttttctgatgcgttgataagcagggagtgattcaaagttaattcagagaagaagagattat |
