# Supplementary material for: Temporal and spatial expression of polygalacturonase gene family members reveals divergent regulation during fleshy fruit ripening and abscission in the monocot species oil palm
Source: BMC Plant Biol. 2012 Aug 25;12:150. doi: 10.1186/1471-2229-12-150 (PMC3546427; doi:10.1186/1471-2229-12-150)
Supplement: Additional file 2 — Standard errors for Figure 3. Percentages were calculated from gene expression data derived from qPCR analysis that included individual values (3 technical repetitions) compared to the average expression of the reference gene (EgEF1α, elongation factor 1 α), together with the standard deviation (SD) for the following three tissue regions of the fruit: AZ, Abscission Zone; M, Mesocarp; P, Pedicel. [file 1471-2229-12-150-S2.doc]

|  | | Gene | Fruit tissue, expression values, percentages and standard deviations | | | | | |
| --- | --- | --- | --- | --- | --- | --- | --- | --- |
|  |  |  | AZ | | M | | P | |
|  |  |  | **Means** | SD | **Means** | SD | **Means** | SD |
| Ethylene Treatment Time (hours) | 0 | *EgPG1* | **0.01** | 0.003 | **0.06** | 0.02 | **0.10** | 0.10 |
|  |  | *EgPG3* | **0.71** | 0.03 | **5.65** | 0.27 | **7.29** | 0.38 |
|  |  | *EgPG4* | **94.32** | 2.38 | **4.37** | 0.38 | **7.11** | 0.96 |
|  |  | *EgPG7* | **0.11** | 0.01 | **0.88** | 0.22 | **1.15** | 0.11 |
|  |  | *EgPG8* | **0.27** | 0.02 | **9.95** | 0.90 | **10.84** | 0.41 |
|  |  | *EgPG9* | **0.26** | 0.01 | **3.88** | 0.53 | **7.28** | 0.90 |
|  |  | *EgPG10* | **1.26** | 0.02 | **15.04** | 1.88 | **20.01** | 1.05 |
|  |  | *EgPG11* | **1.55** | 0.12 | **42.68** | 2.56 | **10.82** | 1.03 |
|  |  | *EgPG16* | **0.13** | 0.01 | **1.96** | 0.16 | **3.23** | 0.12 |
|  |  | *EgPG17* | **0.02** | 0.002 | **3.41** | 0.53 | **4.29** | 0.43 |
|  |  | *EgPG18* | **1.21** | 0.07 | **9.58** | 0.66 | **24.46** | 0.98 |
|  |  | *EgPG19* | **0.03** | 0.004 | **0.82** | 0.15 | **1.20** | 0.12 |
|  |  | *EgPG22* | **0.03** | 0.01 | **1.35** | 0.05 | **1.32** | 0.19 |
|  |  | *EgPG26* | **0.09** | 0.01 | **0.37** | 0.06 | **0.92** | 0.21 |
|  |  | TOTAL | **100.00** | 2.70 | **100.00** | 8.37 | **100.00** | 7.01 |
|  |  |  |  |  |  |  |  |  |
|  | 6 | *EgPG1* | **0.002** | 0.0003 | **0.002** | 0.001 | **0.11** | 0.03 |
|  |  | *EgPG3* | **0.04** | 0.003 | **0.04** | 0.002 | **3.21** | 0.10 |
|  |  | *EgPG4* | **99.33** | 2.85 | **98.64** | 4.54 | **4.23** | 0.21 |
|  |  | *EgPG7* | **0.01** | 0.001 | **0.01** | 0.0004 | **0.58** | 0.03 |
|  |  | *EgPG8* | **0.01** | 0.002 | **0.004** | 0.0004 | **0.20** | 0.02 |
|  |  | *EgPG9* | **0.06** | 0.003 | **0.11** | 0.01 | **7.03** | 0.32 |
|  |  | *EgPG10* | **0.43** | 0.02 | **0.99** | 0.04 | **61.60** | 1.95 |
|  |  | *EgPG11* | **0.05** | 0.002 | **0.17** | 0.01 | **18.63** | 0.38 |
|  |  | *EgPG16* | **0.01** | 0.001 | **0.01** | 0.0005 | **0.42** | 0.01 |
|  |  | *EgPG17* | **0.002** | 0.001 | **0.003** | 0.001 | **0.57** | 0.10 |
|  |  | *EgPG18* | **0.04** | 0.003 | **0.02** | 0.001 | **2.83** | 0.27 |
|  |  | *EgPG19* | **0.002** | 0.0001 | **0.003** | 0.0002 | **0.12** | 0.02 |
|  |  | *EgPG22* | **0.005** | 0.001 | **0.004** | 0.001 | **0.13** | 0.04 |
|  |  | *EgPG26* | **0.02** | 0.001 | **0.01** | 0.001 | **0.33** | 0.05 |
|  |  | TOTAL | **100.00** | 2.70 | **100.00** | 4.60 | **100.00** | 3.54 |

**Supplementary Table 1.**

Percentages were calculated from gene expression data derived from qPCR analysis that included individual values (3 technical repetitions) compared to the average expression of the reference gene (*EgEF1α *: elongation factor 1 α), together with the standard deviation (SD) for the following three tissue regions of the fruit: AZ, Abscission Zone; M, Mesocarp; P, Pedicel.
