# Supplementary figures and images for: Temporal and spatial expression of polygalacturonase gene family members reveals divergent regulation during fleshy fruit ripening and abscission in the monocot species oil palm
Source: BMC Plant Biol. 2012 Aug 25;12:150. doi: 10.1186/1471-2229-12-150 (PMC3546427; doi:10.1186/1471-2229-12-150)

## Slide 1
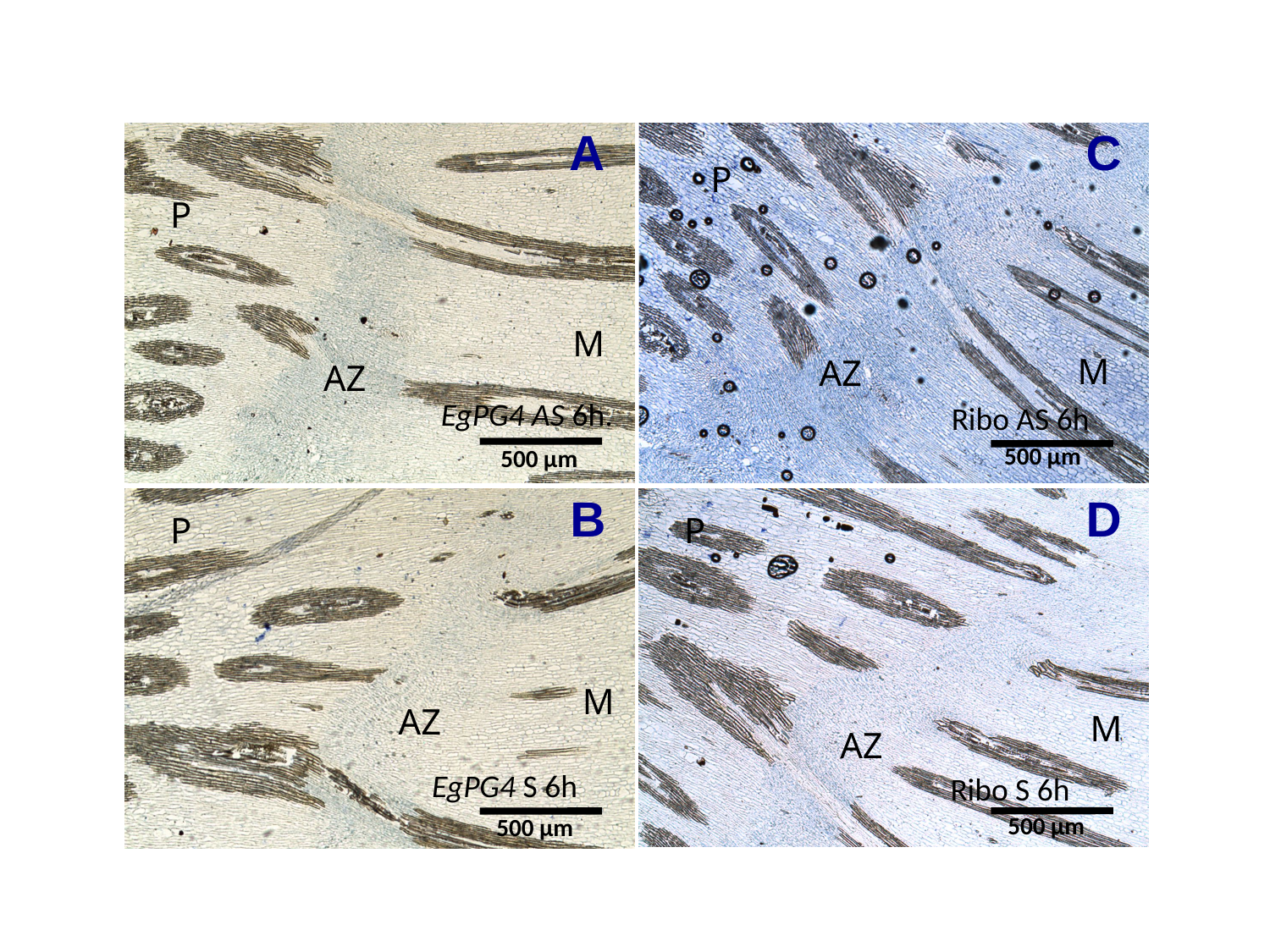

A
C
P
P
M
M
AZ
AZ
EgPG4 AS 6h.
Ribo AS 6h
500 µm
500 µm
B
D
B
P
P
M
AZ
M
AZ
EgPG4 S 6h
Ribo S 6h
500 µm
500 µm

Supplement: Additional file 3 — Control experiments for in situ hybridization studies. Longitudinal sections of the fruit base were hybridized with digoxigenin-labelled RNA fragments of EgPG4 antisense (A) and sense (B), and the 18S ribosome antisense (C) and sense (D) probes after 6h ethylene treatment. [file 1471-2229-12-150-S3.ppt]

## Slide 1
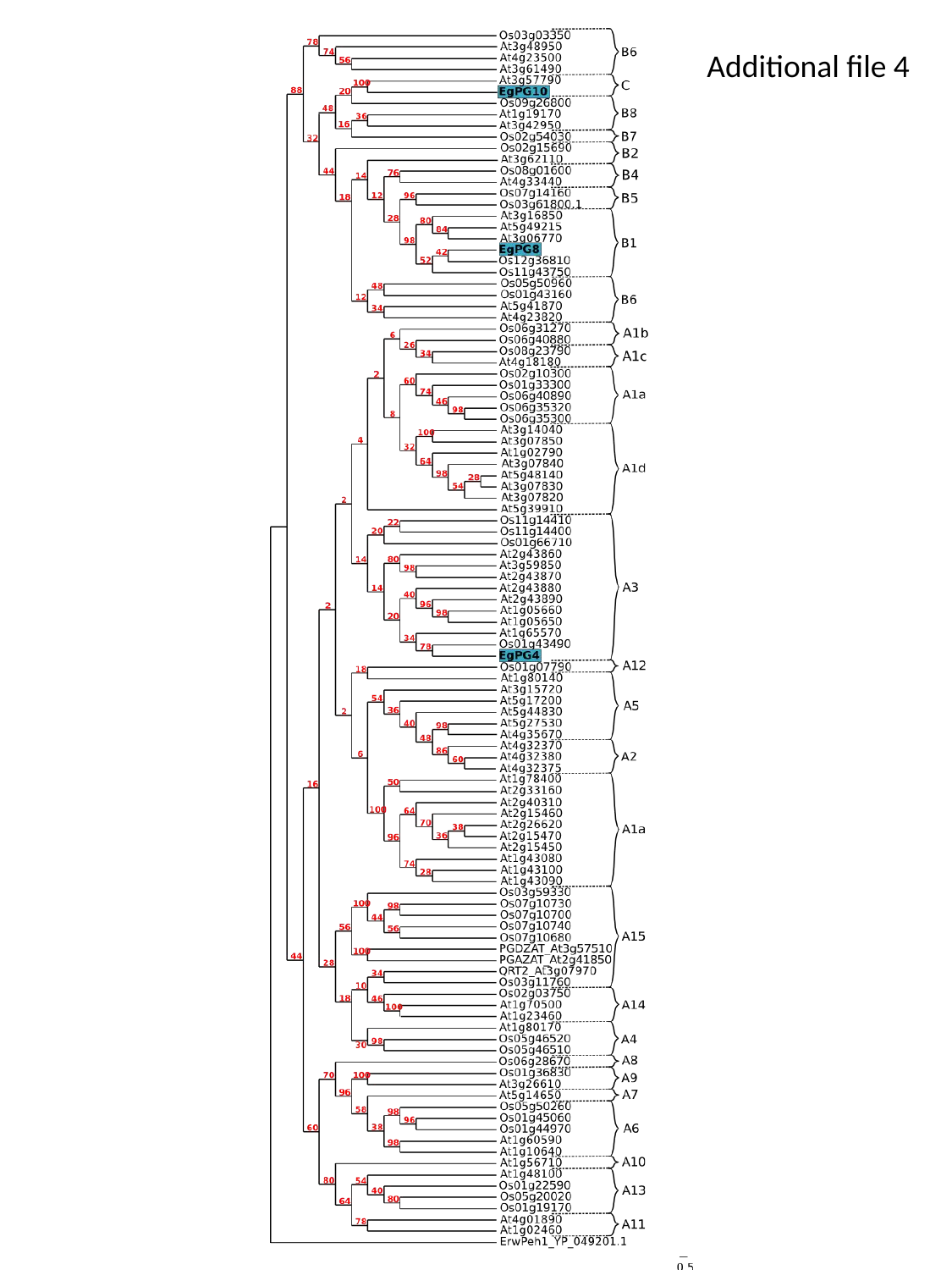

Additional file 4

Supplement: Additional file 4 — Phylogenetic analysis of EgPG4, EgPG8 and EgPG10 with sequences from Arabidopsis and rice. [file 1471-2229-12-150-S4.ppt]
