# Supplementary material for: Temporal and spatial expression of polygalacturonase gene family members reveals divergent regulation during fleshy fruit ripening and abscission in the monocot species oil palm
Source: BMC Plant Biol. 2012 Aug 25;12:150. doi: 10.1186/1471-2229-12-150 (PMC3546427; doi:10.1186/1471-2229-12-150)
Supplement: Additional file 5 — List of primers used for expression analysis of oil palm PG genes by qPCR. [file 1471-2229-12-150-S5.doc]

**Table S1 List of primers for the expression analysis of oil palm PG genes by qPCR.**

| **Name of genes** | **Name and sequence of primers** |
| --- | --- |
| ***EgPG1*** | **EGPG1qS1** ACAAACAGAGCTAAAGACCC  **EGPG1qAS1** GGTGCACAAATATACTGAAACTAC |
| ***EgPG3*** | **EGPG3qS1** GTTACCCAGCTAATCATTTGAAAC  **EGPG3qAS1** CAAACATTTCGGACAAATGAAGA |
| ***EgPG4*** | **EGPG4qS1** ACCTACGGAAACAAGCC  **EGPG4qAS1** AATCCTACATCACCCATTTCA |
| ***EgPG7*** | **EGPG7qS1** CTCATCCAAGCTCCAATATTAGT  **EGPG7qAS1** TTTATTCTGATACCATGCTTTGAGTTA |
| ***EgPG8*** | **EGPG8qS1** AGCTACATTTCTTCATTCAAACTGTAA  **EGPG8qAS1** CTCCTATCCCATTCCCAGATAA |
| ***EgPG9*** | **EGPG9qS1** AATACTGATGGCATTGATCCAGA  **EGPG9qAS1** GAGACCCTGCGAACAAC |
| ***EgPG10*** | **EGPG10qS1** CACAAGATACTATGATCCTTCGT  **EGPG10qAS1** TTTCTAAGAAGTCCACCACCG |
| ***EgPG11*** | **EGPG11qS1** Aatctccgaggttgcattc  **EGPG11qAS1** AACCTTCAAGGCTTTAACATTT |
| ***EgPG16*** | **EGPG16qS1** CATGTCAGAATCTCGACCTTT  **EGPG16qAS1** ACTGCCACGGAATAACC |
| ***EgPG17*** | **EGPG17qS2** TGTGGATGTTGTGAAGTCGAA  **EGPG17qAS2** ATGGGTAAAGAACCTGTTGG |
| ***EgPG18*** | **EGPG18qS2** GTTGTCACATCAAACACTCTGGTA  **EGPG18qAS2** CCTCTGCTCCGTTCAAT |
| ***EgPG19*** | **EGPG19S1** TGAGCTATTGAAGCAGACAC  **EGPG19AS1** ATTTATGAAGCATGGACAAATACG |
| ***EgPG22*** | **EGPG22qS1** CATTGTGAATGCTAGCTCAATTAT  **EGPG22qAS1** CATTGGTAGTGCCTGTAAGT |
| ***EgPG26*** | **EGPG26qS2** AAAGGATGTCTCATACATGAACATAAA  **EGPG26qAS2** GGGTCATCCACCAACTC |
| ***EgEF1*** | **EGEF1S4** TATCAAAGGATGGGCAGACC  **EGEF1AS4** TCATCATACCTTGCCTTGGA |
