# Supplementary material for: Temporal and spatial expression of polygalacturonase gene family members reveals divergent regulation during fleshy fruit ripening and abscission in the monocot species oil palm
Source: BMC Plant Biol. 2012 Aug 25;12:150. doi: 10.1186/1471-2229-12-150 (PMC3546427; doi:10.1186/1471-2229-12-150)
Supplement: Additional file 6 — List of primers used for the synthesis of in situ hybridation probes. [file 1471-2229-12-150-S6.doc]

**Table S2 List of primers used for the synthesis of *in situ* hybridization probes.**

| **Name of genes** | **Name and sequence of primers** |
| --- | --- |
| ***EgPG4*** | **EGPG4qS1** ACCTACGGAAACAAGCC  **EGPG4qS1T7** GAAATTAATACGACTCACTATAGGGAGAACCTACGGAAACAAGCC  **EGPG4qAS1** AATCCTACATCACCCATTTCA  **EGPG4qAS1T7** GAAATTAATACGACTCACTATAGGGAGAAATCCTACATCACCCATTTCA |
| ***EgRIB*** | **EGRiboS** GCCGACCCTGATCTTCTG  **EGRiboST7** GAAATTAATACGACTCACTATAGGGAGAGCCGACCCTGATCTTCTG  **EGRiboAS** TCTTTCGCCCCTATACCCAA  **EGRiboAST7** GAAATTAATACGACTCACTATAGGGAGATCTTTCGCCCCTATACCCAA |
